# Flavonol and A-type procyanidin-rich extracts of *Prunus spinosa* L. flower exhibit anticoagulant activity through direct thrombin inhibition, but do not affect platelet aggregation *in vitro*

**DOI:** 10.3389/fphar.2023.1307373

**Published:** 2023-11-27

**Authors:** Anna Marchelak, Joanna Kolodziejczyk-Czepas, Michał B. Ponczek, Oleksandra Liudvytska, Magdalena Markowicz-Piasecka, Beata Bielska, Katarzyna Miłowska, Monika A. Olszewska

**Affiliations:** ^1^ Department of Pharmacognosy, Faculty of Pharmacy, Medical University of Lodz, Lodz, Poland; ^2^ Department of General Biochemistry, Faculty of Biology and Environmental Protection, University of Lodz, Lodz, Poland; ^3^ Department of Applied Pharmacy, Faculty of Pharmacy, Medical University of Lodz, Lodz, Poland; ^4^ Department of General Biophysics, Faculty of Biology and Environmental Protection, University of Lodz, Lodz, Poland

**Keywords:** *Prunus spinosa*, blackthorn flowers, polyphenols, hemostasis, thrombin, anticoagulant, blood platelets

## Abstract

**Background:** Blackthorn flower (*Prunus spinosa* L.) is a traditional herbal remedy recommended for treating cardiovascular diseases (CVDs).

**Aim:** This *in vitro* study investigates the effects of flavonol and A-type procyanidin-rich blackthorn flower extracts on the hemostatic system, including the blood plasma coagulation cascade and platelet aggregation.

**Methods:** Six distinct extracts, characterized through various techniques, including LC-MS/MS, were assessed at *in vivo*-relevant levels (1–50 μg/mL) for their antithrombotic activity. The thrombin, prothrombin, and activated partial thromboplastin times were measured. Additionally, the thrombin enzymatic activity was tested using the chromogenic substrate S-2238 and fibrinogen as the physiological substrate of the enzyme. To gain insights into the mechanism of action, the interactions between the primary extracts’ constituents, their potential metabolites, and thrombin were examined *in silico*. The computational analyses were complemented by *in vitro* experiments and circular dichroism spectroscopy. The platelet aggregation in human platelet-rich plasma was assessed after ADP or collagen stimulation. Furthermore, the extracts’ biocompatibility was tested on human peripheral blood mononuclear cells (PBMCs) and red blood cells (RBCs).

**Results:** The extracts slightly prolonged the prothrombin and thrombin times and effectively inhibited the thrombin’s enzymatic activity, reducing its amidolytic and proteolytic functions at 50 μg/mL by 91.2% and 74.8%, respectively. *In silico* molecular docking demonstrated a strong binding affinity of the examined polyphenols and their metabolites to thrombin. Most analytes bound exclusively within the enzyme active site; however, afzelin, kaempferitrin, and procyanidin A2 revealed the affinity to additional binding sites, including exosite I. The structure-activity relationship of flavonols as thrombin inhibitors was studied *in vitro*. Circular dichroism spectroscopy confirmed that the interactions between thrombin and the compounds (even at 1 μg/mL) induce alterations within the α-helices’ secondary structure, resulting in noticeable changes in the enzyme’s CD spectrum. On the other hand, the extracts did not influence platelet aggregation. Eventually, their cellular biocompatibility with PBMCs and RBCs was confirmed.

**Conclusion:** The extracts directly inhibit thrombin, a critical serine protease in hemostasis and a prime anticoagulant drug target, and do not exhibit antiplatelet effects. This study enhances the knowledge of the biological activity of blackthorn flowers and supports their traditional use in CVDs.

## 1 Introduction

The physiological balance among platelets, plasma coagulation factors, and fibrinolytic proteins is vital for maintaining blood fluidity, preventing blood loss during injury and controlling the extent of the formed fibrin clot. This equilibrium results from the interplay of procoagulant and anticoagulant elements in the blood, along with the modulatory functions of the vessel wall, particularly the endothelium. However, multiple endogenous and exogenous factors can disrupt this balance, increasing plasma procoagulant activity and reducing fibrinolysis efficiency. Inflammation, elevated plasma fibrinogen levels, platelets hyperactivity, and prothrombotic events are common in various disorders, including cardiovascular diseases (CVDs) (Petäjä, 2011; Stark and Massberg, 2021).

Thrombin (the coagulation factor IIa, EC 3.4.21.5), an executive serine protease of the blood coagulation cascade, plays a central role in forming fibrin clots from plasma fibrinogen. Beyond this, it has various procoagulant and modulatory functions, including activating other coagulation factors and stimulating platelets (Al-Amer, 2022). Due to its critical role in hemostasis, thrombin is a target for anticoagulant medications (Sun et al., 2020). Common thrombin-inhibiting drugs include traditional anticoagulants, which inhibit free thrombin indirectly by binding simultaneously to antithrombin and exosite II, and direct thrombin inhibitors (DTIs). DTIs is a relatively new class of anticoagulants, specifically and potently blocking thrombin by binding to its active site (univalent DTIs) or both active site and exosites (bivalent DTIs), and not dependent on a cofactor such as antithrombin (Lee and Ansell, 2011; Sun et al., 2020). They offer clinical advantages such as more predictable anticoagulant effect, inhibition of thrombin-induced platelet aggregation, and a lower risk of immune-mediated thrombocytopenia. However, they still have limitations and adverse effects, among which the risk of bleeding is the most concerning (Lee and Ansell, 2011). Exploring new, safer thrombin inhibitors, potentially from natural sources, is a promising approach to prevent and treat thromboembolic diseases.

Polyphenols are specialized plant metabolites with pleiotropic activity in cardiovascular system, including antioxidant, anti-inflammatory, antiplatelet and anti-hypertensive effects (Behl et al., 2020). They are able to modulate activity or even inhibit various enzymes, including serine proteases (Xue et al., 2017). Recent studies have noted polyphenols’ ability to counteract thrombin action, particularly for isolated compounds like flavonols, anthocyanidins, flavonolignans, and flavan-3-ols (Liu et al., 2010; Bijak et al., 2014). However, there are limited reports on the thrombin-inhibitory effects of polyphenol-rich extracts (Bijak et al., 2013; Sikora et al., 2014; Wei et al., 2019; Rutkowska et al., 2021). Considering the observed synergy between various types of polyphenols and the potential advantages of using extracts over isolated compounds, the search for polyphenol-rich extracts with antithrombotic properties is justified.

Blackthorn (*Prunus spinosa* L.) is a spiny, deciduous shrub or small tree native to Europe, western Asia, and northwest Africa, with naturalized population in New Zealand, Tasmania and North America. In numerous countries, this plant is not only recognized for its edible fruits but also revered for centuries as a medicinal plant. Ethnopharmacological investigations have revealed long-standing use of blackthorn-derived herbal materials (flowers, fruits, leaves, branches, bark) in folk medicine for various purposes, including application in cardiovascular diseases (Vokou et al., 1993; Cavero et al., 2011; Calvo and Cavero, 2014; Ginko et al., 2023). Flowers are especially valued in European tradition for their vasoprotective, anti-inflammatory, diuretic, detoxifying (blood purifying), and spasmolytic properties, and recommended as ingredients of compound herbal prescriptions traditionally applied, e.g., to treat myocarditis, cardiac neurosis and atherosclerosis (Berger, 1949; Wawrzyniak, 1992; Vokou et al., 1993). Our previous research in chemical, enzymatical and biological models *in vitro* documented that polyphenol-rich blackthorn flower extracts exhibit potent antioxidant and anti-inflammatory activity that might be responsible for the effects indicated by traditional medicine (Marchelak et al., 2017; Marchelak et al., 2019; Marchelak et al., 2021). In particular, the extracts were able to scavenge reactive oxygen/nitrogen species (ROS/RNS) considered as the potential inducers of oxidative stress in the circulatory system *in vivo*, they enhanced the total antioxidant status of human plasma, as well as protected the human plasma biomolecules against oxidative and nitrative impairments (Marchelak et al., 2017; Marchelak et al., 2019). Furthermore, significant protection against ROS/RNS-driven structural and functional modification of fibrinogen, a multifunctional protein essential for hemostasis, was observed (Marchelak et al., 2021). However, the influence of blackthorn flower extracts on the hemostatic system, including the verification of the anticoagulant or antiplatelet properties, has not been investigated.

Therefore, this study aimed to analyze the effects of six distinct *P. spinosa* flower extracts (thoroughly characterized using various phytochemical methods, including LC-MS/MS) on the hemostasis, with a particular emphasis on their potential antithrombotic action. The anticoagulant properties of the extracts were assessed *in vitro* by measuring blood plasma clotting times, as well as thrombin’s enzymatic activity, including both amidolytic and proteolytic functions. To gain insights into the molecular-level mechanism of action, *in silico* analyses were conducted to examine the interaction between the primary constituents of *P. spinosa* extracts, their potential plasma metabolites, and thrombin. In addition to computational analyses, this study included *in vitro* experiments and circular dichroism spectroscopy. Furthermore, to gain a deeper understanding of the extracts’ influence on the hemostatic system, their antiplatelet effects were investigated. In all analyses, the extracts were used at microgram concentrations (*i.e.* 1-50 μg/mL), providing physiologically achievable levels of their bioactive compounds. The final aspect of our study focused on assessing the safety of the *P. spinosa* extracts on human peripheral blood mononuclear cells (PBMCs) and human red blood cells (RBCs).

## 2 Materials and methods

### 2.1 Plant material and preparation of the extracts

The analyses were carried out using dry extracts obtained from the flowers of *P. spinosa* L. Commercial samples of the plant material were bought in 2015 (harvested in April 2015) from a local Polish provider, *Dary Natury* (Koryciny, Poland). The voucher sample (01052015/PSFL_1501_DN) was deposited in the Department of Pharmacognosy, Medical University of Lodz, Poland. The source aqueous extract (AQ) was prepared as follows: the plant material was grounded using an electrical grinder, sieved (0.315 mm), and extracted with chloroform (3 L, 30 h) in a Soxhlet apparatus. Then, the pellet was extracted exhaustively with water (4 × 1 L), and the combined extracts were evaporated *in vacuo* and lyophilized (Alpha 1–2/LD Plus freeze dryer, Christ, Osterode am Harz, Germany). The preparation of the basic methanol-water (7:3, *v/v*) extract (MED) and its concentrated polyphenol-rich fractions of diethyl ether (DEF), ethyl acetate (EAF), *n*-butanol (BF) fractions and water residues (WR), obtained by sequential liquid-liquid partitioning, was described previously (Marchelak et al., 2017).

### 2.2 Phytochemical standardization of the extracts

The qualitative profiling (UHPLC-PDA-ESI-MS^3^ analysis) of AQ was performed according to (Marchelak et al., 2017) using the same equipment and chromatographic procedure. The quantitative profiling of AQ included the measurements of the total phenolic contents (TPC) by the Folin-Ciocalteu method, the total proanthocyanidin contents (TPA) by *n*-butanol-HCl method, as described previously (Marchelak et al., 2017), and the contents of individual analytes by HPLC-PDA assay, performed according to (Marchelak et al., 2020b), using the same equipment and procedure. MED, DEF, EAF, BF and WR were assessed qualitatively and quantitatively in terms of their polyphenolic constituents using a panel of fully validated phytochemical profiling methods, including LC-MS/MS and LC-PDA assays, as was described earlier (Marchelak et al., 2017; Marchelak et al., 2020a; Marchelak et al., 2020b).

### 2.3 Biological material

The study was approved by the Committee on the Ethics of Research at the Medical University of Lodz (RNN/213/18/KE, RNN/104/20/KE) and the University of Lodz (8/KBBN-UŁ/II/2015). All the experiments were performed in accordance with the Declaration of Helsinki and based on the national legal procedures and the European Union regulations.

Blood plasma was obtained by differential centrifugation of the commercially available buffy coat units, constituting the waste of the blood-derived preparations and purchased from the Regional Centre of Blood Donation and Blood Treatment in Lodz (Poland). Fibrinogen was isolated from human plasma as described previously (Marchelak et al., 2021), and its concentration was established spectrophotometrically at 280 nm using an extinction coefficient of 1.55 for 1 mg/mL solution. For the aggregometry tests, fresh blood was obtained from healthy volunteers and collected into the S-Monovette^®^ 8.5 mL, CPDA1 tubes (Sarstedt AG & Co. KG, Nümbrecht, Germany). PBMCs were isolated from human blood using the Histopaque®-1077 medium, according to the previously described procedure (Matczak et al., 2018). The material for RBCs lysis assay and microscopy studies constituted the blood obtained from the Regional Centre of Blood Donation and Blood Treatment in Lodz (Poland). RBCs were separated from plasma by centrifugation (3000 rpm, 10 min, 20°C), washed three times with 0.9% saline, and used for the studies within 24 h (Markowicz-Piasecka et al., 2015).

### 2.4 Determination of the hemostatic parameters

When determining the hemostatic parameters, stock solutions of the *P. spinosa* flower extracts (10 mg/mL) were prepared in 30% DMSO. DMSO concentrations in working solutions of the tested extracts (0.1, 0.5 and 5 mg/mL) were 15%. The studies mixed 5 µL of the extracts with 495 µL of blood plasma or thrombin solution. Control samples were untreated with the extracts; however, they contained a vehicle for the plant preparations, i.e., DMSO. The effect of DMSO on the investigated parameters was excluded in preliminary tests. All experiments used argatroban, a reference anticoagulant drug, as a positive control.

#### 2.4.1 Blood clotting times

Thrombin (TT), prothrombin (PT) and the activated partial thromboplastin time (APTT) were measured in blood plasma using a Kselmed K-3002 Optic coagulometer (Kselmed, Grudziadz, Poland) and reagents purchased from Diagon Kft. (Budapest, Hungary), according to the laboratory protocols delivered by the manufacturer. Before the assays, the blood plasma samples were pre-incubated with the examined extracts at the final 1–50 μg/mL concentration for 15 min at 37°C. Control samples were prepared using blood plasma untreated with the extracts.

#### 2.4.2 Amidolytic activity of thrombin

The thrombin enzyme, purchased from Biomed (Lublin, Poland), was diluted with 0.05 M Tris-buffered saline (TBS), pH 7.4, to the concentration of 0.75 U/mL, and pre-incubated with the examined extracts at the final concentration of 1–50 μg/mL for 10 min, at 37°C. In control samples, 20% DMSO instead of the extracts was used. The measurements were conducted using a kinetic protocol, with absorbance recorded at 415 nm, every 10 s, for 15 min. The reaction mixture comprised 280 µL of thrombin (pre-incubated with the extracts or control/untreated enzyme) and 40 µL of 3 mM chromogenic substrate Chromogenix S-2238 purchased from Instrumentation Laboratory (Bedford, MA, United States of America). Thrombin amidolytic activity was estimated using the BMG Labtech software based on the maximal velocity of the reaction (V_max_). The assays were performed using 96-well microplates and the BMG Labtech SpectroStar Nano spectrophotometer (BMG LABTECH, Offenburg, Germany).

Analogous pre-incubation conditions and experimental systems were used to determine effects of the several components of the extracts (selected based on the results of the phytochemical profiling and molecular docking study), i.e., quercetin (QU), kaempferol (KA), quercetin 3-*O*-*α*-L-arabinofuranoside (avicularin, AV), quercetin 3-*O*-*α*-L-rhamnopyranoside (quercitrin, QC), kaempferol 3-*O*-*α*-L-arabinofuranoside (juglanin, JU), kaempferol 3-*O*-*α*-L-rhamnopyranoside (afzelin, AFZ), kaempferol 3,7-di-*O*-*α*-L-rhamnopyranoside (kaempferitrin, KT), kaempferol 3-*O*-(*2″*-*O*-E-*p*-coumaroyl)-α-L-arabinofuranoside, *p*-cJU), proanthocyanidin A2 (PA2), and 5-*O*-caffeoylquinic acid (chlorogenic acid, CHA) on the amidolytic activity of the enzyme. High-purity standards of QU and CHA were purchased from Sigma-Aldrich (St. Louis, MO, United States), while KA and PA2 were obtained from Phytolab (Vestenbergsgreuth, Germany). The standards of AV, QC, JU, AFZ, KT, *p*-cJU were isolated previously in the Department of Pharmacognosy, Medical University of Lodz, Lodz, Poland, from the flowers and leaves of *P. spinosa*, with HPLC and NMR purity >98% (Olszewska and Wolbis, 2001; Olszewska, 2002; Olszewska and Wolbiś, 2002).

#### 2.4.3 Proteolytic activity of thrombin

The proteolytic activity of thrombin was determined using fibrinogen isolated from human blood plasma, as was described in section 2.3. The reaction mixture consisted of 100 µL fibrinogen (diluted with TBS to the concentration of 3 mg/mL) and 200 µL thrombin (0.75 U/mL in TBS enriched with 25 mM CaCl_2_, control or pre-incubated with the tested extracts, analogously to the measurements of the amidolytic activity). Kinetic measurements were started immediately after adding thrombin to the microplate wells with fibrinogen. The absorbance was recorded at 360 nm, every 10 s, for 20 min, at 37°C. The proteolytic activity of thrombin was estimated using BMG Labtech software (SpectrostarNano Mars, Version 3.01. R2) based on the maximal velocity of the polymerization reaction (V_max_) and lag time. The assays were conducted using 96-well plates, and a microplate reader SpectrostarNano (BMG Labtech, Ortenburg, Germany). The effects of the extracts on the proteolytic activity of thrombin were also evaluated using human blood plasma. The experiments were carried out analogously to the abovementioned procedure, using 100 µL of human blood plasma instead of the isolated fibrinogen.

### 2.5 *In silico* molecular docking

The selected constituents of *P. spinosa* flower extracts, including CHA, 3- *O*-caffeoylquinic acid (neochlorogenic acid, NCHA), (+)-catechin (CA), PA2, QU, KA, AV, JU, QC, *p*-cJU, AFZ, kaempferol 3-*O*-*α*-L-arabinofuranoside-7-*O*-*α*-L-rhamnopyranoside (KAFR), KT, kaempferol 3-*O*-*β*-D-xylopyranoside (KX), kaempferol 3-*O*-(4″-*O*-*β*-D-glucopyranosyl)-*α*-L-rhamnopyranoside (multiflorin B, MUB), kaempferol 7-*O*-*α*-L-rhamnopyranoside (KR), *p*-coumaric acid (*p*-CA), as well as phenolic compounds considered to be primary metabolites of polyphenols in the human body, including miquelianin (quercetin 3-*O*-*β*-D-glucuronopyranoside, MQ), 3-(3′,4′-dihydroxyphenyl)propionic acid (dihydrocaffeic acid, DCA), protocatechuic acid (PCA), 3-(4′-hydroxyphenyl)propionic acid (PPA), 2-(3′,4′-dihydroxyphenyl) acetic acid (PAA) were prepared for docking to the unliganded (3U69 structure) and liganded (1FPH.PDB structure) macromolecule of human thrombin as described earlier (Kolodziejczyk-Czepas et al., 2017). The docking was accomplished using AutodockVina 1.1.2 (Trott and Olson, 2010), ten times for each ligand. Average and standard deviations (SD) of the predicted ten-fold binding affinity (kcal/mol) were taken for further evaluation. The docked molecular structures were illustrated using UCSF Chimera 1.13 (http://www.cgl.ucsf.edu/chimera/, Pettersen et al., 2004).

### 2.6 Circular dichroism (CD)

The CD spectra (195–260 nm) were recorded for human thrombin purchased from Sigma-Aldrich (St. Louis, MO, United States), in the presence/absence of the selected constituents of *P. spinosa* flower extracts (PA2, KA, *p*-cJU, QU) on a Jasco J-815 CD spectropolarimeter (Tokyo, Japan) in 5-mm path length quartz cuvettes, with a wavelength step of 1 nm, a response time of 4 s and a scan rate of 100 nm/min, thermostatted at 37°C. Each spectrum was the average of three repetitions. Human thrombin was used at a 15 μg/mL concentration in 10 mM phosphate buffer (pH = 7.4). In determining circular dichroism spectra, stock solutions of *P. spinosa* flower components (10 mg/mL) in 10% tetrahydrofuran were initially prepared. The final concentration of tetrahydrofuran in the sample was less than 0.05%. The selected extract components in phosphate buffer without thrombin at the concentrations used in the experiments were used as a baseline for thrombin/extract complexes spectra.

### 2.7 Blood platelet aggregation

The blood samples under examination contained a normal range of blood platelets, ranging from 2.15 × 10^5^ to 3.41 × 10^5^ cells/μl. Platelet-rich plasma (PRP) was prepared by differentially centrifuging whole blood according to the method described earlier (Kolodziejczyk-Czepas et al., 2021). Subsequently, freshly obtained PRP samples (495 μL) were pre-incubated for 15 min at 37°C with the tested analytes (5 μL). These mixtures were then transferred into Chrono-Log 490 aggregometer cuvettes (Havertown, United States). Platelet aggregation measurements were conducted after stimulating the samples with either ADP (a final concentration of 10 μM) or collagen (a final concentration of 2 μg/mL), following the manufacturer’s instructions.

### 2.8 Determination of the cellular safety

#### 2.8.1 Influence on PBMCs viability

The cytotoxicity of the examined extracts was performed in an experimental model of PBMCs, according to the procedure described earlier (Matczak et al., 2018). PBMCs were isolated from human blood, suspended in the RPMI-1640 medium (1.5 × 106 PBMCs/mL) and incubated with the extracts at the final concentration of 1–50 μg/mL for 24 h. Cell viability measurements were carried out using the resazurin-based metabolic assay (*In Vitro* Toxicology Assay Kit, Sigma-Aldrich) according to the procedure described by the manufacturer.

#### 2.8.2 Influence on erythrocyte membrane integrity/also hemolysis

Microscopy studies were conducted as follows: a 2% RBCs suspension in 0.9% saline was incubated at 37°C for 1 or 24 h with examined extracts at the final concentration of 1–50 μg/mL. Afterwards, erythrocyte suspension was vigorously mixed, and morphology was evaluated using a phase contrast Opta-Tech inverted microscope (Opta-Tech, Warszawa, Poland), at 400-times magnification, equipped with software (OptaView 7) for image analysis. RBC lysis assay was conducted spectrophotometrically by measuring the amount of hemoglobin released from RBCs, according to (Markowicz-Piasecka et al., 2015). Briefly, RBCs suspension (2% in 0.9% saline) was incubated for 1 and 24 h at 37°C with the examined extracts at the final 1–50 μg/mL concentration. After the incubation, the samples were centrifuged (1000 rpm, 10 min), and the absorbance of the supernatant was measured at 550 nm. A sample of 2% v/v Triton X-100 purchased from Polish Chemical Reagents (Gliwice, Poland) represented 100% of hemoglobin release (positive control), whereas the sample of 0.9% saline represented spontaneous hemolysis of RBCs (negative control).

### 2.9 Statistical analysis

The results are presented as mean values ±standard error (SE) or standard deviation (SD) for the indicated number of experiments. The significance of the differences between means was evaluated using a one-way ANOVA or one-way ANOVA for repeated measurements, followed by the *post hoc* Tukey’s test for multiple comparisons or *post hoc* Dunnett’s test. The correlations were determined using an *F*-test. All calculations were performed using the Statistica 12 PL software for Windows (StatSoft Inc., Krakow, Poland). The *p* values lower than 0.05 were considered significant.

## 3 Results

### 3.1 Phytochemical profiling

The analyses were performed using primarily the source aqueous (AQ) and hydroalcoholic (MED) extracts prepared from the flowers of *P. spinosa* L. Moreover, the concentrated polyphenol-rich fractions (DEF, EAF, BF) and water residues (WR) obtained by fractionated extraction of MED were also included.

**FIGURE 1:**
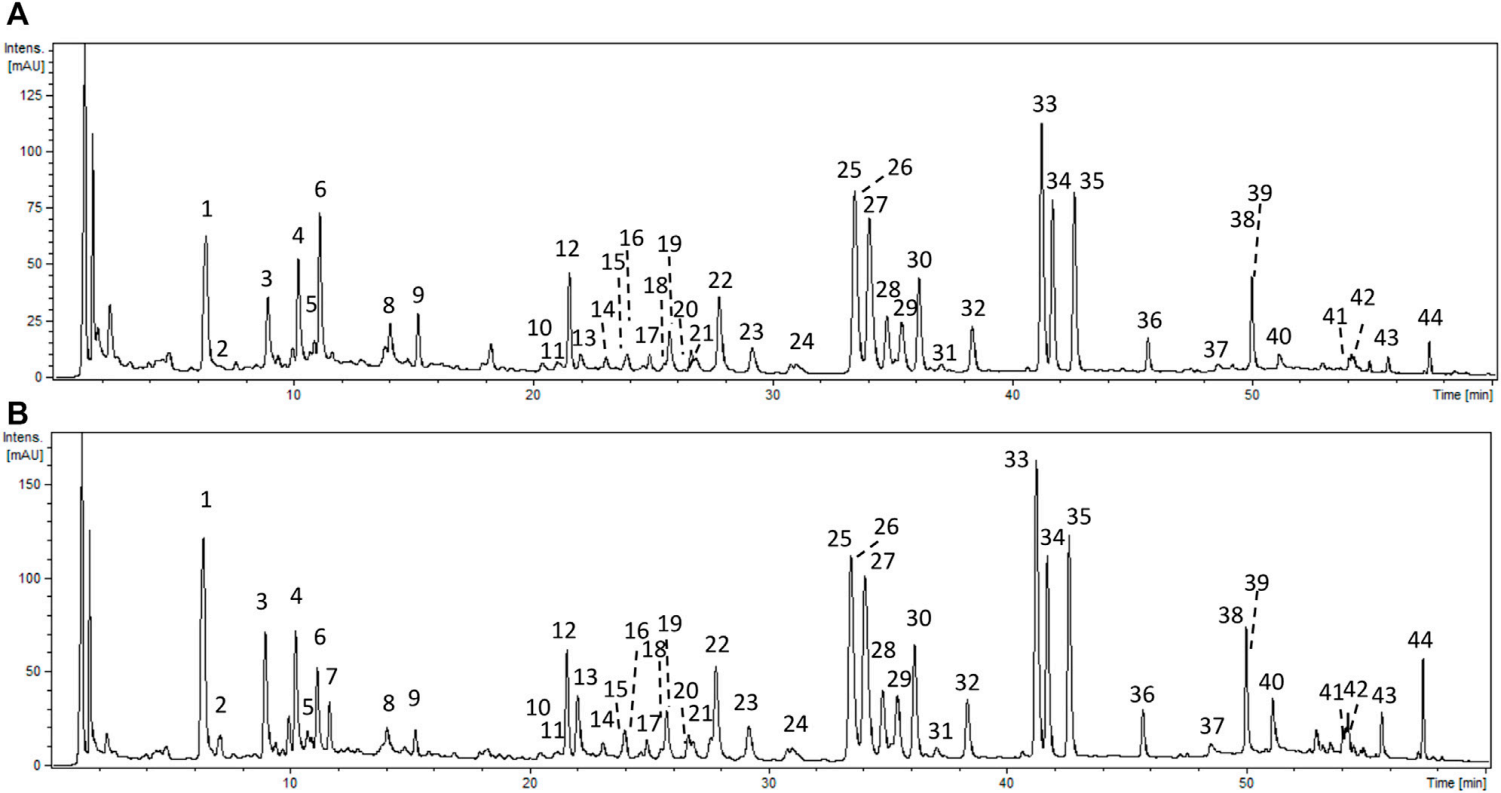
Representative UHPLC chromatograms of *P. spinosa* aqueous extract (AQ) **(A)** and hydroalcoholic (methanol-water, 7:3, *v/v*) (MED) **(B)** extracts at 280 nm. Peak numbers refer to those implemented in Supplementary Table S1.

The UHPLC-PDA-ESI-MS^3^ analysis of AQ enabled the detection of 43 constituents representing different phenolic classes: flavonols (kaempferol and quercetin glycosides), flavan-3-ols (catechins and A-type proanthocyanidins), and phenolic acids (Figure 1; Supplementary Table S1). The analytes were structurally characterized based on comparing their chromatographic retention and spectral profiles (UV-Vis, ESI-MS^3^) with the literature data or reference standards, both commercial and isolated previously in our laboratory from flowers and leaves of *P. spinosa* (Olszewska and Wolbis, 2001; Olszewska, 2002; Olszewska and Wolbiś, 2002). The total phenolic content determined by the Folin-Ciocalteu assay (TPC) was 126.59 mg of gallic acid equivalents (GAE) per g of dry weight (dw), while the total content of the individual analytes determined by the RP-HPLC-PDA method (TPH) amounted to 77.71 mg/g dw. Among the compounds with the highest levels (>5 mg/g dw) are kaempferol diglycosides, such as KT, KAFR, and MUB, as well as kaempferol and quercetin monoglycosides, including AV and JU. The total proanthocyanidin content (TPA) determined by *n*-butanol assay was 24.46 ± mg CYE/g dw (Table 1).

**TABLE 1:** Quantitative profile of the source extracts obtained from *P. spinosa* flower (mg/g dw).

Analyte	Content (mg/g dw)	
	AQ	MED
Individual compounds		
**NCHA**	4.89 ± 0.14^*B*^	14.46 ± 0.23^*A*^
**CHA**	3.83 ± 0.07^*B*^	5.64 ± 0.11^*A*^
**CA**	< LOQ	< LOQ
**CCHA**	4.51 ± 0.06^*A*^	4.26 ± 0.07^*B*^
**CFA**	< LOQ	< LOQ
**ECA**	< LOQ	< LOQ
**KAPR**	< LOQ	2.69 ± 0.10
**LEP**	1.21 ± 0.04^*B*^	3.17 ± 0.09^*A*^
***p*-CA**	< LOQ	< LOQ
**KT**	5.65 ± 0.06^*B*^	17.42 ± 0.79^*A*^
**KAFR**	5.15 ± 0.10^*B*^	15.13 ± 0.21^*A*^
**RT**	2.53 ± 0.14^*B*^	4.65 ± 0.15^*A*^
**QGA**	4.80 ± 0.06^*B*^	6.28 ± 0.25^*A*^
**IQ**	0.41 ± 0.03^*B*^	1.33 ± 0.03^*A*^
**HY**	0.74 ± 0.03^*B*^	0.92 ± 0.04^*A*^
**KRG**	1.22 ± 0.12^*B*^	3.67 ± 0.14^*A*^
**RN + GU**	2.26 ± 0.09^*B*^	4.26 ± 0.15^*A*^
**MUA**	3.71 ± 0.04^*B*^	5.38 ± 0.20^*A*^
**AV**	8.59 ± 0.11^*B*^	14.89 ± 0.65^*A*^
**QC**	3.37 ± 0.09^*B*^	7.41 ± 0.21^*A*^
**KX**	1.73 ± 0.05^*B*^	2.97 ± 0.10^*A*^
**MUB**	6.63 ± 0.10^*B*^	8.82 ± 0.07^*A*^
**JU**	8.61 ± 0.15^*B*^	13.73 ± 0.43^*A*^
**AFZ**	5.65 ± 0.15^*B*^	13.33 ± 0.16^*A*^
**KR**	0.60 ± 0.06^*B*^	1.78 ± 0.04^*A*^
**KCAR**	< LOQ	1.47 ± 0.02
**QU**	0.57 ± 0.03^*B*^	1.32 ± 0.06^*A*^
**KA**	0.72 ± 0.02^*B*^	1.06 ± 0.01^*A*^
***p*-cJU**	0.35 ± 0.02^*B*^	1.43 ± 0.03^*A*^
Phenolic fractions		
**TPH**	**77.71**	**157.47**
**TPC**	**126.98 ± 1.61** ^***B***^	**206.07 ± 10.86** ^***A***^
**TPA**	**24.46 ± 1.26** ^***B***^	**45.13 ± 2.38** ^***A***^

The results are presented as means ± SD (*n* = 3). Different superscripts in each row indicate significant differences in the means at *p* < 0.05. The quantitative profile of MED, according to (Marchelak et al., 2017; Marchelak et al., 2020a; Marchelak et al., 2020b).

The bold values refer to the content of groups of compounds not individual constituents.

The same panel of phytochemical profiling methods was used previously for the standardization of MED, DEF, EAF, BF and WR (Marchelak et al., 2017; Marchelak et al., 2020a; Marchelak et al., 2020b). The comparison of the recorded UHPLC profiles revealed that qualitative profiles of the source extracts AQ and MED are almost identical (Figure 1; Supplementary Table S1). However, the quantitative profiles of the extracts vary significantly. The concentrations of phenolics and proanthocyanidins were about 1.6–2 times higher for MED than for AQ. Among the individual compounds, KT, KAFR, MUB, AV and JU were the most abundant in both extracts, still reaching 1.6–3 times higher levels in MED than in AQ (Table 1). The summary of the results from the quantitative studies for all the extracts/fractions used in the present investigation was given in Supplementary Table S2.

### 3.2 Blood clotting tests and determination of the thrombin enzymatic activity

To determine the impact of *P. spinosa* extracts on the hemostatic activity of blood plasma, the well-known diagnostic parameters, i.e., blood clotting times such as TT, PT and aPTT, were used (Table 2). A slight but statistically significant PT prolongation was observed for BF and AQ (about 4.7% and 3.5% at 50 μg/mL, respectively). Moreover, MED, DEF, BF and WR moderately increased TT (by 3.4%–5.1% at 50 μg/mL) with statistical significance.

**TABLE 2:** Determination of the effects of *P. spinosa* flower extracts on blood clotting times of human plasma. Results are presented as means ± SE (*n* = 12). Statistical differences: **p < 0.01, ***p < 0.001 for samples in the presence of the analytes (50 μg/mL) or the reference inhibitor, argatroban (0.5, 5, 10 μg/mL) *versus* control samples.

*P. spinosa* extracts or a reference anticoagulant drug	Concentration [µg/mL]	Clothing times [% of the control/untreated human plasma]		
		PT	APTT	TT
MED	50	100.37 ± 0.95	101.85 ± 0.95	103,36 ± 0.84**
DEF	50	100.02 ± 0.75	100.36 ± 0.99	104,03 ± 0.76**
EAF	50	100.56 ± 0.68	100.61 ± 0.98	101,40 ± 0.82
BF	50	104.67 ± 1.33**	98.94 ± 0.61	105,12 ± 1.30***
WR	50	102.29 ± 1.08	99.31 ± 0.74	104,89 ± 1.02***
AQ	50	103.49 ± 1.11**	100.36 ± 1.15	99,27 ± 1.46
Argatroban	0.5	134.01 ± 1.50***	202.58 ± 2.32***	974.60 ± 27.50***
	5	562.65 ± 12.69***	423.72 ± 7.83***	No clot
	10	821.54 ± 46.95***	No clot	No clot

**FIGURE 2:**
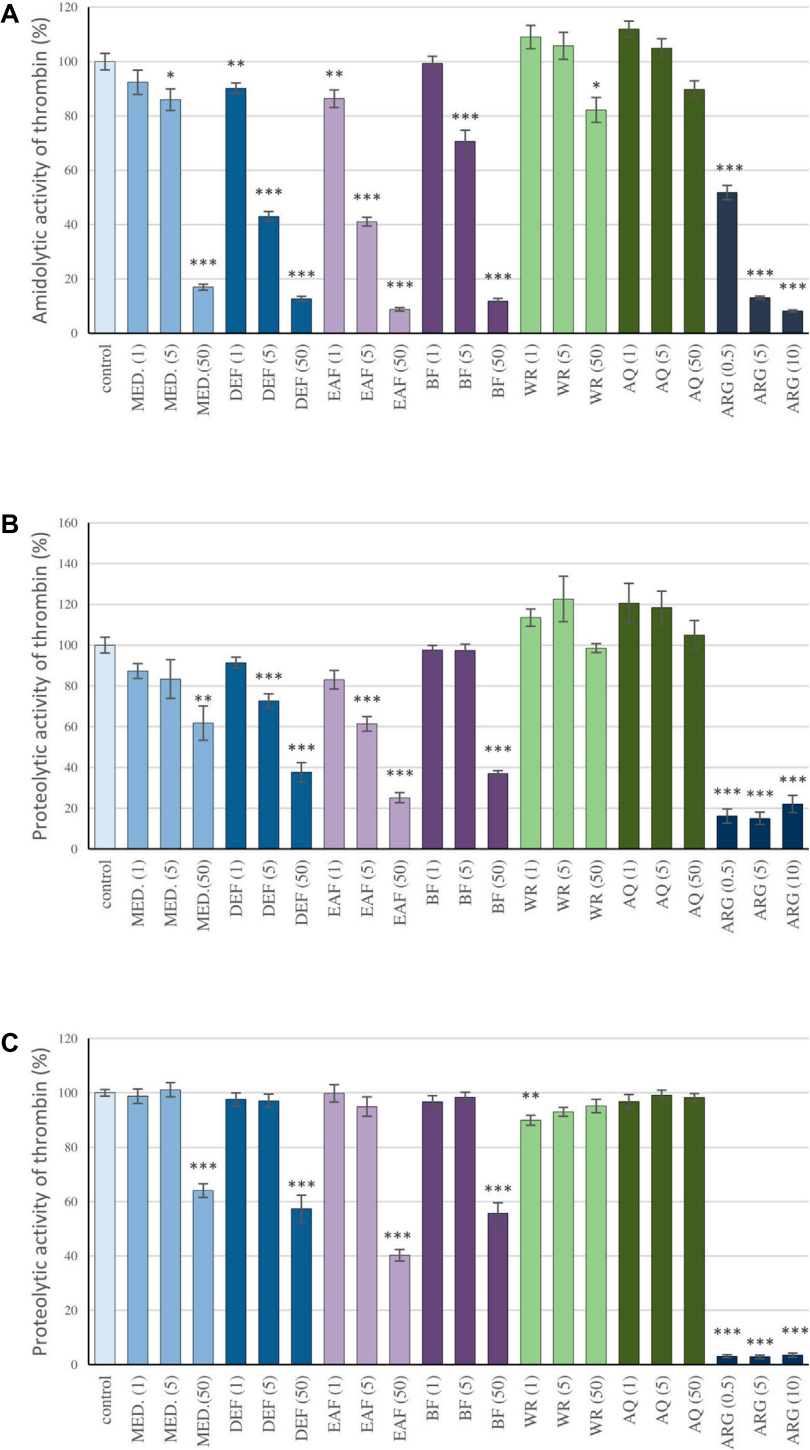
Inhibitory effects of the examined *P. spinosa* flower extracts on the amidolytic **(A)** and proteolytic activity of thrombin **(B)** study on the isolated fibrinogen; **(C)** study on the human plasma). The assays were performer using the kinetic protocol and the TH activity was calculated based on the Vmax parameter. Results are presented as means +SE (n = 6). Statistical differences: * *p* < 0.05, ** *p* < 0.01, and *** *p* < 0.001 for samples in the presence of the analytes (1, 5, 50 µg/mL) or the reference inhibitor, argatroban (0.5, 5, 10 µg/mL) *versus* control samples.

The influence of the extracts on thrombin enzymatic activity was investigated in different experimental systems *in vitro*, including the measurements of the amidolytic and proteolytic activity of the enzyme. The amidolytic activity of thrombin was measured based on the enzyme’s ability to hydrolyze a peptide-*p*NA bond in the chromogenic substrate D-Phe-Pip-Arg-*p*NA. This molecule is patterned after the *N*-terminal portion of the α chain of fibrinogen, the physiological substrate for thrombin. The analyses of the V_max_ parameter revealed that MED and its fractions partly antagonize the reaction in a concentration-dependent manner. DEF and EAF were the most active fractions; even at 1 μg/mL and 5 μg/mL, they inhibited the reaction by about 10% and 60%, respectively, while at 50 μg/mL, the effects reached up to approximately 90%. On the other hand, the efficiency of AQ was lower than MED; at 50 μg/mL, the percentage of inhibition was merely about 10%, and the effect was not statistically significant. (Figure 2A).

The capacity of blackthorn extracts to inhibit the proteolytic activity of thrombin was investigated based on the kinetic studies on the proteolysis of fibrinogen by thrombin and fibrin generation, primarily two key parameters of the polymerization curve: the lag time and the maximum velocity of the process (V_max_). The lag time corresponds to the thrombin-induced cleavage of fibrinopeptides A and B from the fibrinogen molecule, while the V_max_ describes the rate of fibrinogen polymerization. The studies on the isolated fibrinogen revealed that the extracts reduced the rate of fibrinogen polymerization, which was shown as a decrease in the V_max_ parameter (Figure 2B). Similarly to the case of the amidolytic activity studies of thrombin, DEF and EAF presented the highest effectiveness: the V_max_ values at 5 μg/mL and 50 μg/mL decreased by 27.4%–38.6% and 62.3% and 74.82%, respectively. In addition, the significantly prolonged lag time was noticed: 525.7 s and 545.3 s for DEF and EAF at 50 μg/mL, respectively, *versus* 10 s observed for the control (Supplementary Figure S1A). Moreover, the plasma matrix studies confirmed the extracts’ ability to inhibit fibrinogen polymerization (Figure 2C; Supplementary Figure S1B). The decreases in V_max_ for MED and its fraction DEF, EAF and BF at 50 μg/mL were in the range of 35.9%–59.7%; at the same time lag time was prolonged from 13.75 s observed for the control to 78.3–355s. On the other hand, the effects of AQ were negligible.

The correlation studies have been performed to evaluate the contribution of polyphenols to the observed effects of blackthorn flower extracts on the amidolytic and proteolytic activity of thrombin (Table 3). The results revealed that the inhibitory effects of the extracts were firmly phenolic-dependent, with significant correlations (*p* < 0.05) found for total phenolics (TPC and TPH) and total proanthocyanidins (TPA). The effective levels of the phenolic fractions responsible for the thrombin inhibitory effects of the blackthorn flower extracts were estimated from the working extract concentrations of 1, 5, and 50 μg/mL and TPC levels; they amounted to 0.1–0.6, 0.3–2.9 and 3.2–29.2 µg of the phenolics/mL.

**TABLE 3:** Correlation coefficients (*r*) and probability (*p*) values of the linear relationships between phenolic contents of *P. spinosa* flower extracts/fractions and their activity parameters towards thrombin–inhibition of amidolytic activity of thrombin, inhibition of proteolytic activity of thrombin assayed on isolated fibrinogen and on the blood plasma.

*r* (*p*) for	TPC	TPH	TPA
Amidolytic activity of thrombin	0.8172 (0.000)*	0.8128 (0.000)*	0.7876 (0.000)*
Proteolytic activity of thrombin (isolated fibrinogen)	0.8259 (0.000)*	0.8114 (0.000)*	0.7897 (0.000)*
Proteolytic activity of thrombin (blood plasma)	0.9400 (0.000)*	0.8952 (0.000)*	0.9283 (0.000)*

Phenolic content and the activity parameters of the extracts/fractions are according to Table 1, Supplementary Table S2 and Figure 2. The inhibitory effects of the extracts/fractions were calculated based on V_max_ parameter. Asterisks mean statistical significance of the estimated linear relationships (*p < 0.05).

In the abovementioned experiments, the effectiveness of *P. spinosa* flower extracts has been compared to argatroban, the anticoagulant drug that acts by directly inhibiting thrombin. Although the ability of MED and its polyphenol-rich fractions to inhibit thrombin activity was lower than that of argatroban, the results indicated their high potential. For example, the inhibition of the amidolytic and proteolytic activity of the enzyme observed for EAF at 50 μg/mL (29.2 µg of the phenolics/mL) were comparable to argatroban at 10 μg/mL.

### 3.3 *In silico* molecular docking

**FIGURE 3:**
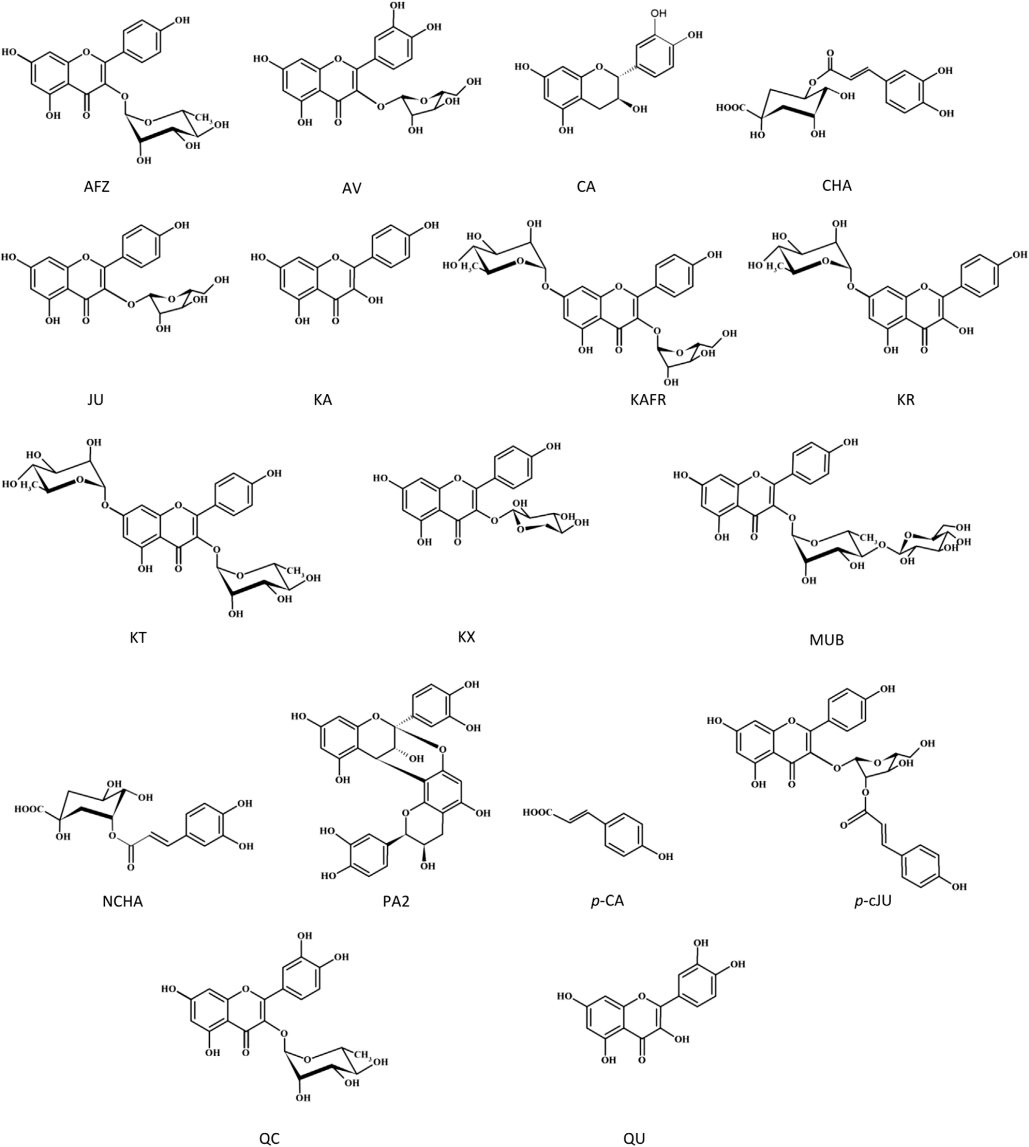
Structures of the tested *P. spinosa* flower extracts constituents.

**FIGURE 4:**
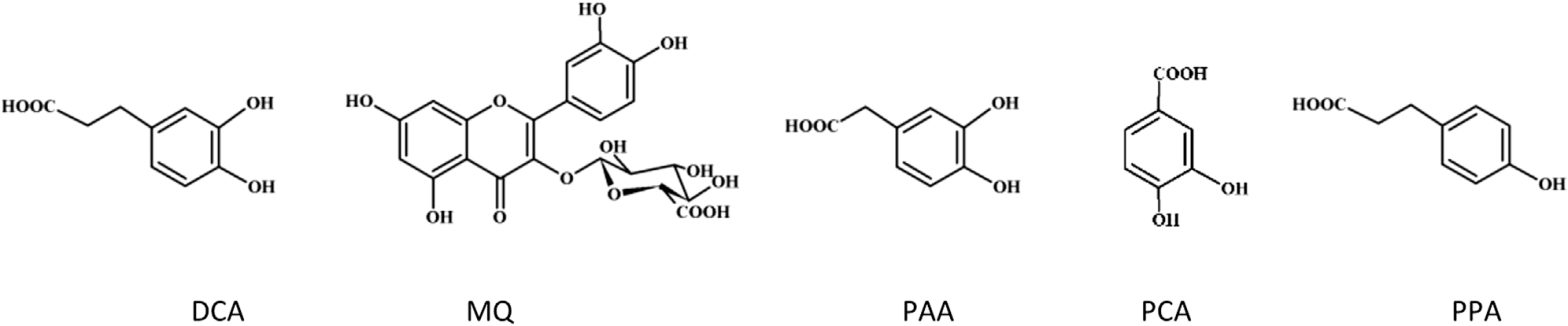
Structures of the tested phenolic metabolites.

**FIGURE 5:**
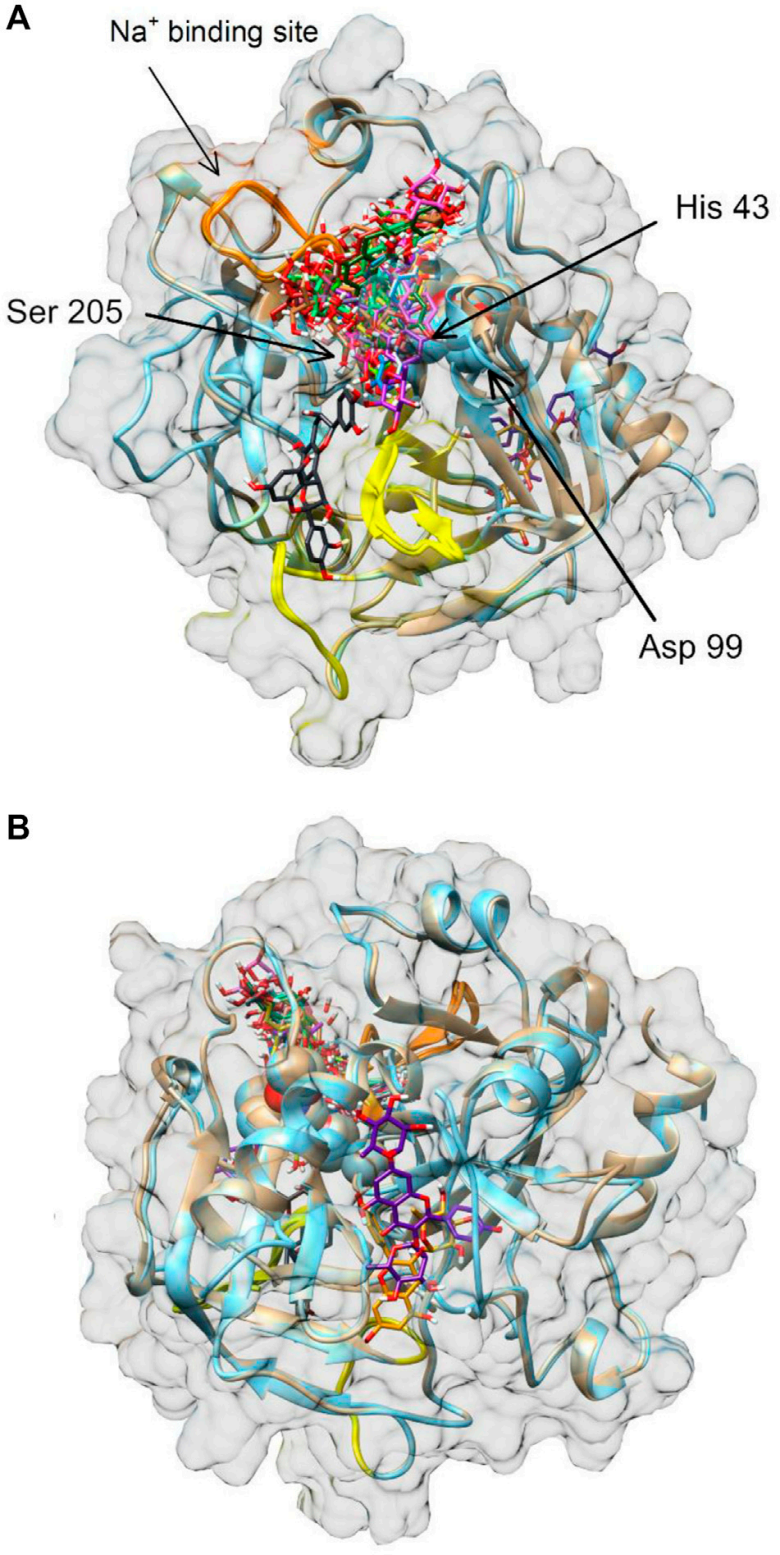
Molecular docking of *P. spinosa* compounds and their potential metabolites *in vivo* to human thrombin. Panel **(A)** front view, panel **(B)** back view. Exosites 1 and 2 are marked in yellow and orange, respectively. Amino acid residues Asp99, His43 and Ser205 of the enzyme catalytic domain (triad active site) are shown as spheres in the center of the thrombin molecule representation. Images of thrombin structures 3U69 and 1FPH are shown in brown and blue, respectively.

The molecular interaction between thrombin and *P. spinosa* polyphenols was analyzed *in silico*. All studied flower constituents (Figure 3) and their potential metabolites *in vivo* (Figure 4) interacted with considerable binding affinity to thrombin within the active site (in unliganded and liganded forms) close to Ser205 and His43 of the catalytic triad (Table 4; Figure 5). Three compounds, i.e., AFZ, KT, and PA2, had additional binding sites outside the catalytic triad of liganded structure; PA2 was bound in exosite I (Figure 5A), while the additional binding of AFZ and KT took place on the opposite side of the thrombin molecule relative to exosite I (Figure 5B).

**TABLE 4:** Binding affinity calculated for constituents of *P. spinosa* flower extracts and their potential metabolites *in vivo* from molecular docking to unliganded and liganded human thrombin. Visualization of protein-compounds complexes–see Figure 5.

	Unliganded 3U69		Liganded 1FPH	
	Average affinity (kcal • mol-1)	± SD	Average affinity (kcal • mol-1)	± SD
Extracts constituents				
AFZ	−7.9	0.0	−8.2	0.6
AV	−8.9	0.2	−8.5	0.2
CA	−8.4	0.0	−8.3	0.6
CHA	−8.1	0.0	−8.0	0.1
JU	−8.4	0.0	−8.2	0.4
KA	−8.2	0.1	−8.0	0.6
KAFR	−8.1	0.5	−8.0	0.3
KR	−9.4	0.0	−9.4	0.0
KT	−8.7	0.0	−8.4	0.4
KX	−9.2	0.0	−8.9	0.1
MUB	−8.3	0.3	−8.3	0.3
NCHA	−7.8	0.1	−8.7	0.0
PA2	−9.2	0.3	−9.0	0.1
*p*-CA	−5.3	0.1	−5.0	0.1
*p*-cJU	−9.9	0.3	−8.7	0.0
QC	−8.0	0.1	−8.6	0.5
QU	−7.4	0.1	−7.5	0.1
Phenolic metabolites				
DCA	−5.6	0.2	−5.3	0.1
MQ	−8.5	0.1	−8.3	0.2
PAA	−5.6	0.2	−5.3	0.1
PCA	−5.5	0.0	−5.3	0.2
PPA	−5.2	0.2	−4.9	0.1

### 3.4 Verification of the thrombin inhibitory effects of *P. spinosa* polyphenols *in vitro*

Based on the phytochemical profiling, *in vitro* evaluation of the extracts’ effects on thrombin activity and *in silico* data, ten compounds (*i.e.,* QU, KA, AV, JU, AFZ, QC, KT, *p*-cJU, PA2 CHA) were selected to verify their inhibitory efficiency towards thrombin *in vitro*. Among them, QU, KA, *p*-cJU and PA2 significantly inhibited the enzymatic activity of thrombin, with the IC_50_ values ranging from 38.29 to 81.12 μg/mL (Table 5). For the remaining six metabolites, no inhibitory effects on thrombin were found at a concentration range of 1–50 μg/mL (data not presented).

**TABLE 5:** The thrombin-inhibitory efficiency of the examined constituents of the *P. spinosa* flower extracts in the amidolytic tests. IC_50_ - the half maximal inhibitory concentration.

Thrombin treated with	IC_50_ [μg/mL]
QU	29.35
KA	81.12
*p*-cJU	38.29
PA2	52.36
Argatroban	<1

### 3.5 Circular dichroism (CD)

**FIGURE 6:**
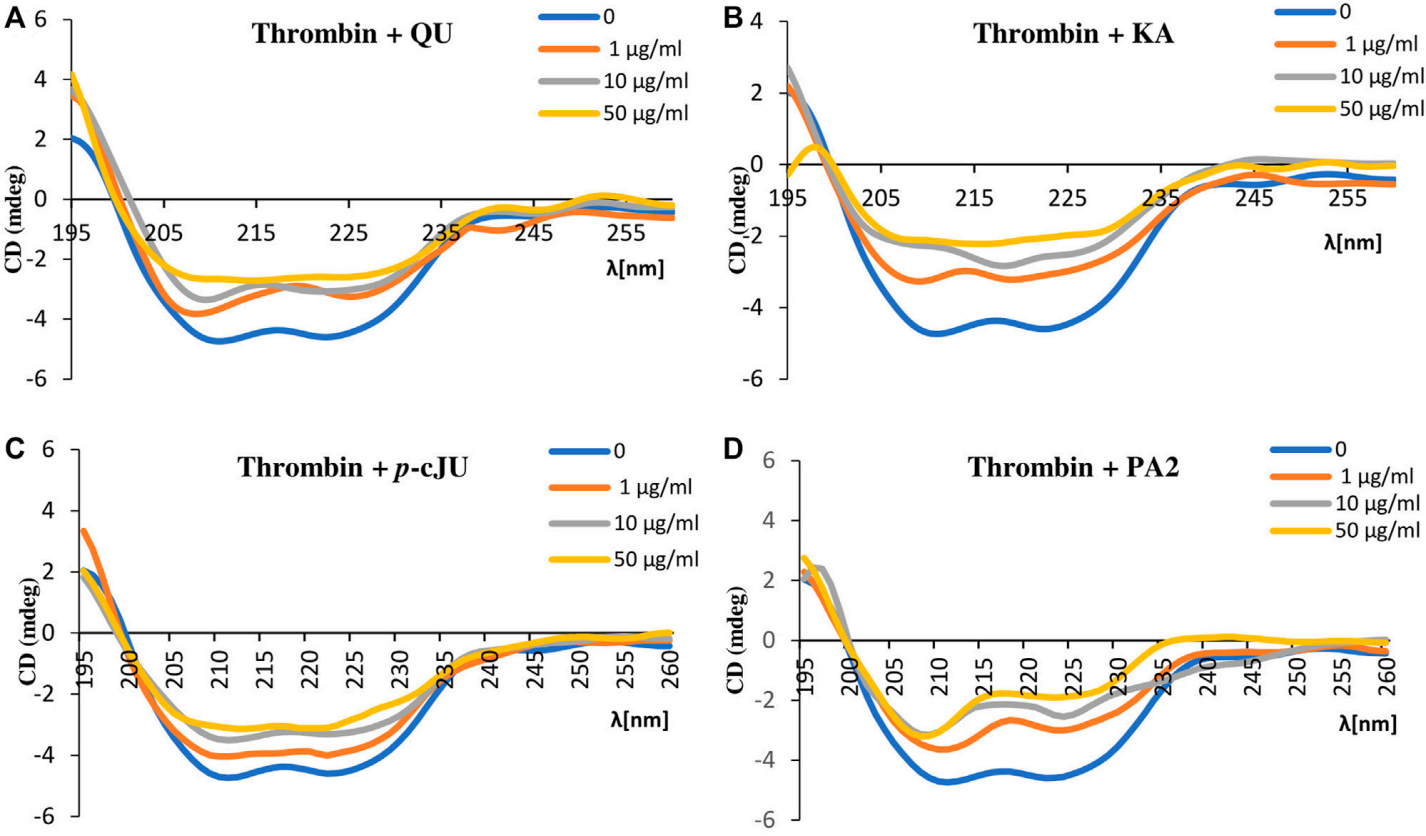
Circular dichroism spectra of thrombin (c = 15 μg/mL) in the presence of selected *P. spinosa* flower extracts constituents: **(A)** QU, **(B)** KA, **(C)** *p*-cJU, **(D)** PA2. Each spectrum is the average of three replicates.

In order to further verify whether the *P. spinosa* polyphenols affect the secondary structure of thrombin, circular dichroism spectra of thrombin alone and in the presence of selected compounds were performed. The thrombin CD spectrum is typical of the *α*-helix polypeptide chain structure with two negative bands centered at 208 nm and 222 nm. The addition of all tested compounds to thrombin resulted in changes in CD spectrum intensity, even at the lowest concentration (1 μg/mL). Higher concentrations of compounds significantly altered the shape of the thrombin CD spectrum, indicating variations in the thrombin secondary structure (Figure 6). The most apparent modification of the thrombin *α*-helix structure has been observed for PA2. Based on the CD spectra, it can be concluded that the selected *P. spinosa* flower constituents changed the secondary structure of thrombin, which may lead to alterations in its enzymatic activity.

### 3.6 Blood platelet aggregation

**FIGURE 7:**
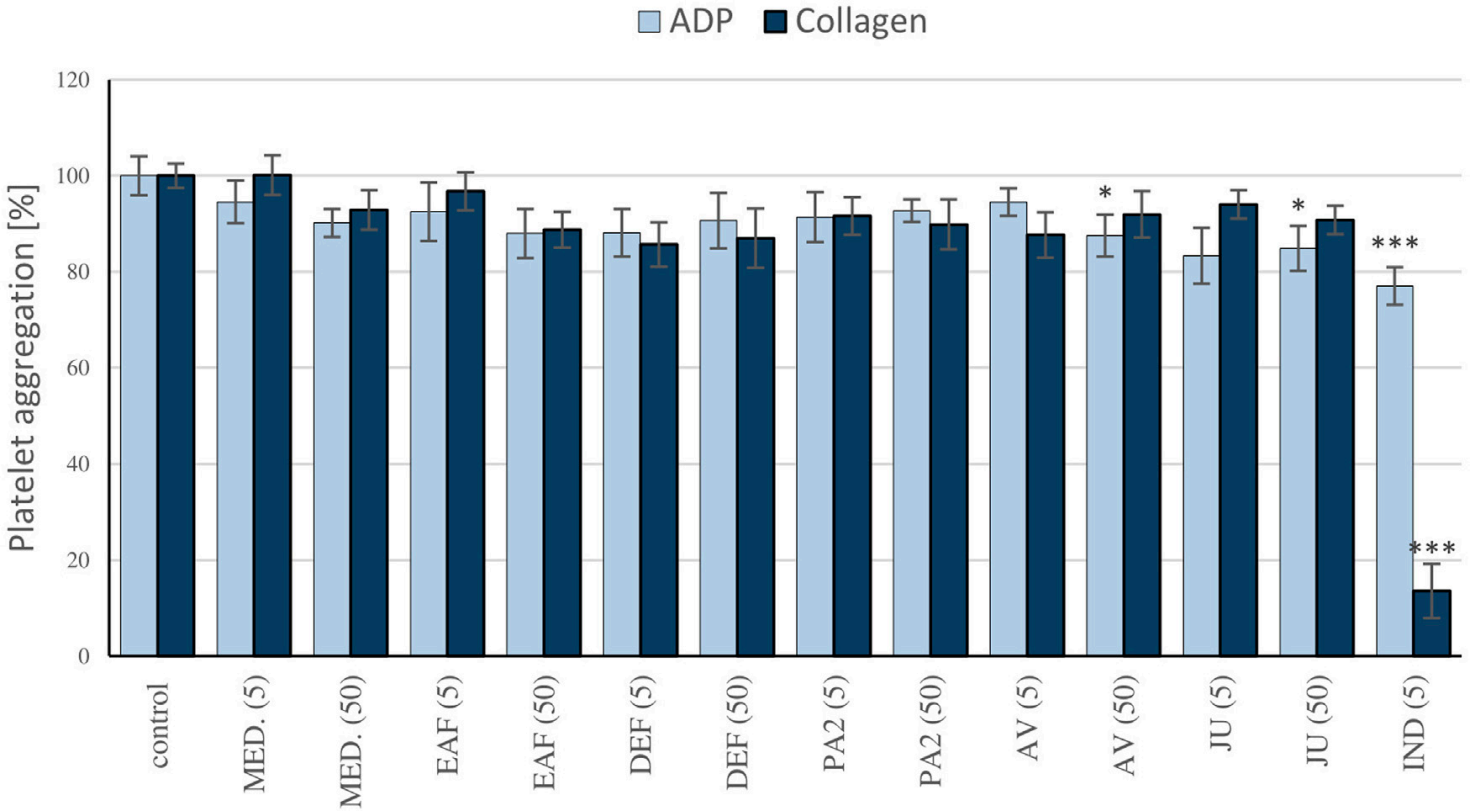
Evaluation of the antiplatelet activity of the *P. spinosa* extracts. The effects of the examined plant extracts on blood platelet aggregation were measured in platelet-rich plasma, following the stimulation by ADP (10 µM) or collagen (2 µg/ml). The aggregation of blood platelets in control platelet-rich plasma samples (not treated with the analytes) was assumed as 100%. Results are presented as means ± SE (*n* = 9). Statistical differences: *p < 0.05, ***p < 0.001 for samples in the presence of the analytes (5, 50 µg/mL) or the reference inhibitor, indomethacin (5 µg/mL) *versus* control samples.

To determine the impact of blackthorn flower extracts on platelet hemostasis, the measurements of ADP and collagen-induced aggregation in PRP were conducted. The study focused on the source extract MED and the fractions EAF and DEF, which consistently demonstrated high potential in prior research. Furthermore, AV, JU, and PA2, identified as the primary compounds in the most active extracts, were selected for the investigation. The results revealed that the extracts (5–50 μg/mL) did not affect platelet aggregation significantly (*p* > 0.05) (Figure 7). Among the tested polyphenols, statistically significant inhibition of platelet aggregation was only observed for AV and JU at a concentration of 50 μg/mL, when ADP was used as the agonist; however, the effects not exceed 15%. Indomethacin, employed as a positive control, at 5 μg/mL inhibited platelet aggregation by approximately 23% and 86% when platelets were stimulated by ADP and collagen, respectively.

### 3.7 PBMCs viability

**FIGURE 8:**
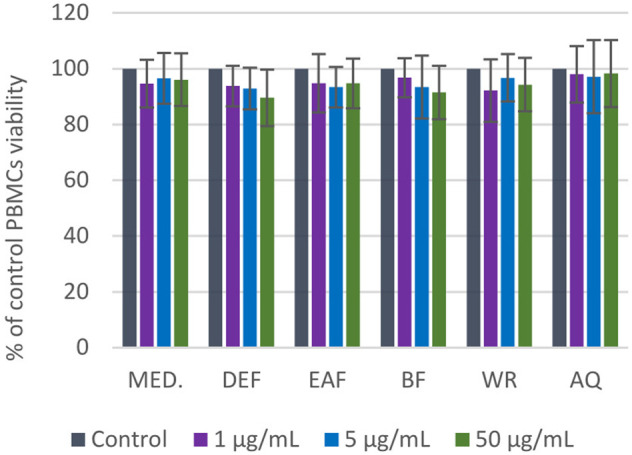
Viability of PBMCs after 24 h of incubation with *P. spinosa* flower extracts at 1-50 µg/mL tested in resazurin-based assay. Results are presented as means +SE (n = 12) for repeated measures. All values are not statistically different (*p* > 0.05).

The potential cytotoxicity of *P. spinosa* extracts/fractions was assessed in the model of PBMCs after 24-h incubation with the extracts at the concentration range of 1–50 μg/mL (Figure 8). The viability of PBMCs treated with the extracts constituted 89.6%–98.3% of the control (untreated) samples. The cellular safety of the extracts was evidenced by the lack of significant differences (*p* > 0.05) between the respective results.

### 3.8 Influence on erythrocyte membrane integrity/also hemolysis

**FIGURE 9:**
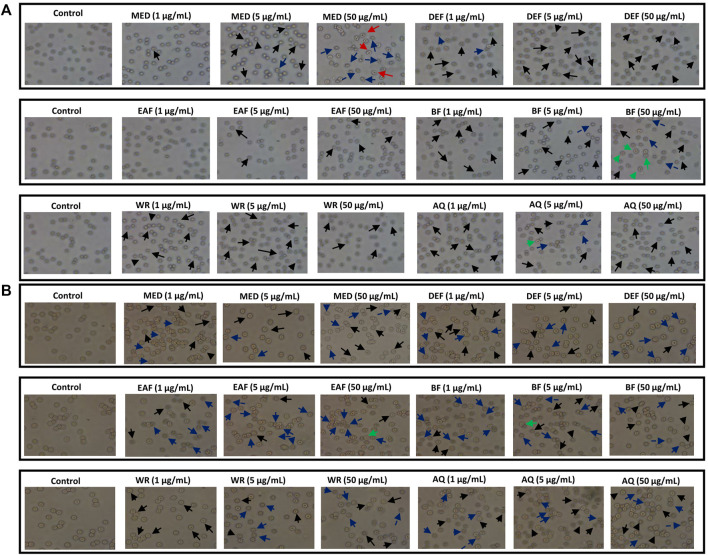
Effects of *P. spinosa* flower extracts at 1, 5 and 50 μg/mL on erythrocytes morphology after 1 **(A)** and 24 h **(B)** incubation. Representative phase-contrast images are shown (magnification of 400 times), echinocytes are marked with black arrows, eryptotic erythrocytes are marked with blue arrows, stomatocytes are marked with red arrows, while ovalocytes with green arrows.

Microscopy studies and hemolysis assay were used to assess the effect of the examined extracts on erythrocyte membrane integrity. At physiological pH 7.4, the erythrocytes suspended in 0.9% saline are primarily observed as discocytes resembling a two-concave disk. After a 1-h co-incubation with the examined extracts/fractions, erythrocytes exhibited a tendency to form echinocytes. These are physiological forms where the discocyte shape is maintained, but the cell membrane folds and forms numerous protrusions (Figure 9A). For instance, DEF, EAF and WR extracts over the entire concentration range contributed to substantial echinocytosis. However, other forms of erythrocytes were detected in the case of other extracts. For example, eryptotic erythrocytes were found after 1-h incubation with MED extract at 5 and 50 μg/mL. In turn, treating RBCs with extract BF at 50 μg/mL resulted in the formation of ovalocytes which are abnormally shaped RBCs. The 24-h incubation of erythrocytes showed that most tested extracts led to extensive echinocytosis (Figure 9B). Furthermore, a trend towards programmed erythrocyte death was observed for all examined extracts (1–50 μg/mL) manifested by a substantial number of eryptotic RBCs. In summary, microscopic analysis showed that examined extracts mainly induce physiological changes in erythrocytes shape, which may indicate that they do not interact strongly or adversely with RBC lipid bilayer.

**FIGURE 10:**
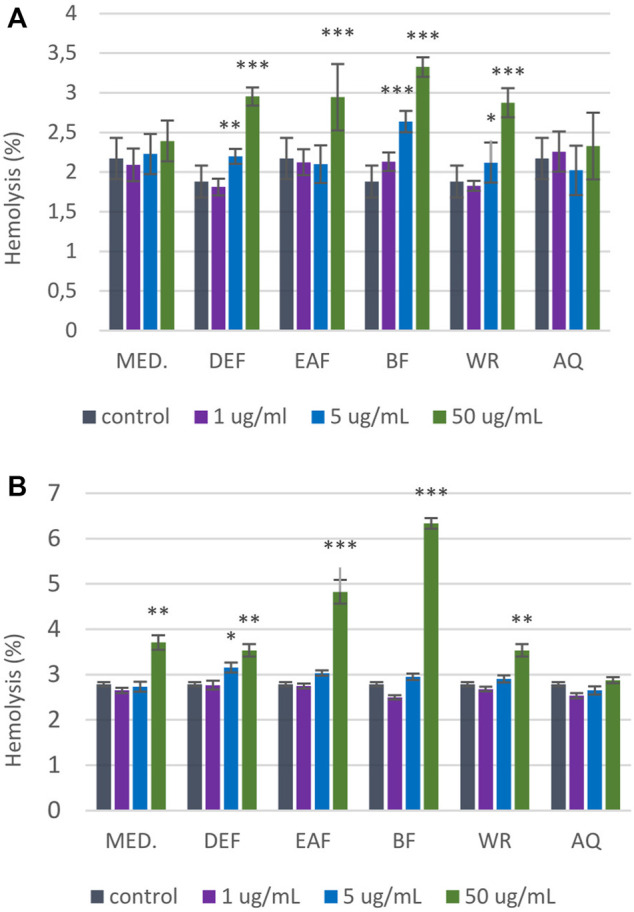
Influence of *P. spinosa* flower extracts on the hemolysis of erythrocytes. The results are presented as percentage of RBCs hemolysis obtained from 1-h **(A)** and 24-h **(B)** interaction of studied extracts with 2% RBCs suspension, compared to the positive control Triton X-100 at 0.2% (100% hemolysis). The results are presented as mean ± SE; n = 5, asterisk denotes significant differences compared to control 0.9% NaCl (*p < 0.05; **p < 0.01; ***p < 0.001).

The results from RBCs lysis assay (Figure 10) showed that AQ over the entire concentration range (1–50 μg/mL) did not contribute to the significant changes in erythrocyte hemolysis after 1-h and 24-h incubation. However, incubation of RBCs with all other extracts at the highest tested concentration (50 μg/mL) significantly increased the percentage of hemolyzed erythrocytes. For instance, DEF at 50 μg/mL contributed to 2.94% ± 0.25% hemolysis after 1-h incubation (*p* < 0.001). More prolonged incubation resulted in 3.53% ± 0.68% of hemolyzed erythrocytes (*p* < 0.01). Notably, in all cases, the degree of RBCs hemolysis accounted for approximately 7%–8%, with BF extract being the most erythrotoxic (6.34% ± 0.57% vs. 2.46% ± 0.34% for control; *p* < 0.001). Since the hemolysis of erythrocytes not exceeding 10% is considered clinically safe (Stasiuk et al., 2009), all examined extracts/fractions can be considered hemocompatible in the 1–50 μg/mL concentration range.

## 4 Discussion

Thrombin is the ultimate trypsin-like serine proteinase formed from the zymogen prothrombin as the final product of activation of both the intrinsic and extrinsic pathways of the blood coagulation cascade (Marchi and López, 2016). The proteolytically-active enzyme has a molecular weight of 37 kDa. It comprises two polypeptide chains: the light chain, formed by 36 amino acids, and the heavy chain, which has 259 amino acids in its structure (Marchi and López, 2016; Al-Amer, 2022). There are four functional domains of thrombin: the active site, the anion binding exosites I and II, and Na^+^ binding site; this unique structure is believed to be responsible for high substrate specificity (Lane et al., 2005; Marchi and López, 2016). The active site of the enzyme, containing the catalytic triad His43, Asp99 and Ser205, is in the central part of the molecule in a negatively charged deep narrow canyon that forms the steric hindrance and restricts access to other molecules. The anion-binding exosites I and II, positively charged regions adjacent to the active site and placed near opposing ends, are also known as the substrate recognition sites as they interact electrostatically with negatively charged groups of the substrates, cofactors, and inhibitors. The Na^+^ binding site, through sodium ions binding, allosterically modulates thrombin activity; the access of the small substrates to the active site increases, and procoagulant substrates are preferred over anticoagulant ones (Crawley et al., 2007; Marchi and López, 2016).

The regulation of thrombin generation and activity is essential for hemostatic equilibrium maintenance and, consequently, one of the main therapeutic strategies for preventing and treating thromboembolic disorders involved in the etiology and pathophysiology of severe CVDs such as myocardial infarction, ischemic stroke, and venous thromboembolic disease. Some plants may be promising sources of substances exerting anticoagulant activities through the inhibition of thrombin. In recent years, the direct interaction with this serine protease has been reported for several plant-derived substances, including polyphenol-rich extracts/fractions from black chokeberry (*Aronia melanocarpa*) (Bijak et al., 2013; Sikora et al., 2014), grape seeds (*Vitis vinifera*) (Bijak et al., 2013), rowanberries (*Sorbus aucuparia*) (Rutkowska et al., 2021), St. John’s Wort (*Hypericum perforatum*) (Wei et al., 2019), alkaloid and polyphenol-rich extracts from *Uncaria tomentosa* leaves and barks (Kolodziejczyk-Czepas et al., 2021), diterpene-rich extracts from *Salviae Miltiorrhizae* roots (Lu et al., 2015; Yang et al., 2020), and bufadienolide-rich fractions from *Kalanchoe daigremontiana* (Kolodziejczyk-Czepas et al., 2017).

In studies of plant-derived substances, carefully selected and exhaustively standardized extracts are crucial to obtain reliable results and drive meaningful conclusions on their bioactivity. As was pointed out in our previous research, the source defatted methanol-water (7:3, *v/v*) extract of *P. spinosa* flower (MED), and its concentrated phenolic fractions obtained by liquid-liquid fractionation (DEF, EAF, BF), appear to be advantageous for functional application. They exhibited a distinct phytochemical profile with a high content of polyphenols, primarily flavonoids, including rare flavonol pentosides, followed by A-type procyanidin dimers, simple phenolic acids, and quinic acid pseudodepsides (Marchelak et al., 2017; Marchelak et al., 2020a; Marchelak et al., 2020b). It is noteworthy that among the substances derived from blackthorn, the flowers appear to be particularly rich in polyphenols. For instance, the total phenolic content (TPC) determined by the Folin-Ciocalteu assay for the dry methanol-water (7:3, *v/v*) extract of flowers was more than twice as high as that for the dry methanol-water (7:3, *v/v*) extract of fresh fruits (206.07 ± 10.86 vs. 87.57 ± 3.54 mg GAE/g dw, respectively). Moreover, in contrast to the flower extracts, distinguished by the remarkable diversity of the flavonoid fraction, in the fruit extracts, phenolic acids and aldehydes are the predominant and structurally diverse group of polyphenols, coexisting with flavonoids and anthocyanins (Marchelak et al., 2017; Magiera et al., 2022). As was pointed out in our previous studies blackthorn flower extracts, *i.e.*, MED and its concentrated fractions, exhibited significant biological effects in complementary *in vitro* models (Marchelak et al., 2017; Marchelak et al., 2019; Marchelak et al., 2021). In particular, they were able to maintain the hemostatic balance of human blood due to the significant protective effects against the oxidative stress-induced structural changes in fibrinogen. Such changes might alter the fibrinogen functions and lead to the generation of dysfunctional hemostatic clots, closely linked with numerous CVDs (Marchelak et al., 2021). Therefore, MED and its fractions have been selected for the present investigation into the impact of *P. spinosa* flower on thrombin inhibition. Moreover, the source aqueous extract (AQ) was included in the study as a representative of herbal teas. Herbal teas, defined as the oral aqueous preparations obtained from plant material through infusion, maceration, or decoction, are the most widely used preparation of traditional medicine and a popular global beverage. Since herbal teas/infusions have gained popularity recently, their composition, efficiency, and safety should be thoroughly evaluated (Poswal et al., 2019; Etheridge and Derbyshire, 2020). Therefore, all the extracts/fractions used in the present study were characterized in detail by applying a panel of phytochemical profiling methods, including LC-MS/MS and LC-PDA assays, fully validated for quantitative purposes. According to the results (Table 1, SSupplementary Table S1, 2; Figure 1), a methanol-water (7:3, *v/v*) provided higher recovery of phenolic compounds than pure water, which is consistent with literature data on the best extraction solvents for low-molecular-weight polyphenols (Brglez Mojzer et al., 2016). Considering the quantitative results, we decided not to fractionate AQ and only include the source extract in the activity and cytotoxic studies, which might allow us to illustrate its bioactivity and safety as a traditional herbal preparation.

The present study indicated for the first time that the extracts/fractions of *P. spinosa* flower contain natural inhibitors of the blood coagulation process. The blood clotting tests revealed a slight prolongation of TT (Table 2). TT is the parameter corresponding to the reaction of the conversion of fibrinogen into fibrin monomers catalyzed by thrombin, and its prolongation occurs in the presence of substances that interfere with fibrin polymerization. The observed tendency to increase TT suggests that blackthorn flower extracts/fractions might act as thrombin inhibitors. Thus, their interaction with the enzyme has been investigated in the next step using various experimental systems *in vitro*, including the low-molecular synthetic substrate and fibrinogen–a physiological target of thrombin–isolated and in a plasma matrix. The tested extracts/fractions have been proven to inhibit amidolytic and proteolytic activity of thrombin even at 1–5 μg/mL (Figure 2; Supplementary Figure S1). MED and its fractions EAF, DEF, and BF revealed the highest capacity of antagonizing thrombin, and they appeared to be the most promising in maintaining the hemostatic balance. On the other hand, the study pointed out that AQ has no anticoagulant effect. These findings show that the extraction from natural matrices is a crucial step in the utilization of phenolic compounds as it affects the composition of the extracts and their biological efficiency. Moreover, as the observed effects strongly correlated with the total levels of phenolics (*p* < 0.05) (Table 3), the studies revealed that the anticoagulant potential of the blackthorn extracts/fractions is phenolic-dependent. What is essential, in all analyses, the extracts/fractions were used in microgram concentrations (1–50 μg/mL), corresponding to 0.1–29.2 μg/mL polyphenols. In case of MED, the concentrations 1, 5, and 50 μg/mL are equivalent to 0.2, 1 and 10 µg of the phenolics/mL, respectively. According to the literature, such levels are physiologically achievable in the blood plasma after oral intake. For instance, the reference plasma level of polyphenols after oral ingestion may reach 3.5–7 μg/mL for quercetin glucosides (Manach et al., 2005) and 2.1–5.2 μg/mL for monocaffeoylquinic acids (Farah et al., 2008).

The results clearly revealed that the extracts/fractions of *P. spinosa* flower, when used at *in vivo*-relevant levels of their active polyphenolic fractions, impact plasma hemostasis. However, they do not significantly affect ADP and collagen-induced platelet aggregation (Figure 7). In the literature, there are several reports on the antiplatelet activity of certain polyphenols, including those present in the investigated extracts. For instance, quercetin has been shown to possess relevant antiplatelet activity by blocking GPIIb/IIIa receptors, suppressing platelet activation, and promoting the pro-aggregate effect of calcium ionophore (Zaragozá et al., 2021). Nonetheless, these effects were observed at a quercetin concentration of 2 mM (approximately 600 μg/mL), which appears unlikely to be achievable *in vivo*. Conversely, the study on kaempferol demonstrated its ability to impair collagen-induced platelet activation through inhibition of NADPH-dependent ROS production at micromolar concentrations (Wang et al., 2015). In the present study, we observed the significant inhibition of blood platelet response to ADP in the presence of quercetin and kaempferol glycosides AV and JU at 50 μg/mL. According to the literature data, glycosylation of flavonols hinders their antiplatelet activity, which might partially explain the only slight anti-aggregatory potential of AV and JU, as well as lack of the effects of the blackthorn extracts rich in flavonols’ glycosides.

The association of computational and experimental methods becomes a popular strategy in identifying novel promising compounds from natural matrices. Among several approaches, molecular docking is a powerful tool for studying interactions between small-molecular ligands and macromolecular targets. It allows to accurately predict the conformation of ligands within the binding site of the target and the ligand-receptor binding free energy (Ferreira et al., 2015; Jakhar et al., 2020). Thus, to indicate the most probable mechanism of the thrombin-inhibitory activity of the *P. spinosa* flower extracts, the experimental studies were supported by *in silico* molecular docking. Based on the phytochemical profiles of the extracts, 17 compounds were selected among leading representatives of all groups of blackthorn polyphenols, including flavonoid aglycones–QU, KA; flavonoid monoglycosides–AV, JU, QC, AFZ, KR, KX, *p*-cJU; flavonoid diglycosides–KAFR, KT, MUB; A-type proanthocyanidins–PA2; flavan-3-ols–CA; caffeoylquinic acids–CHA, NCHA; and simple hydroxycinnamic acids–*p*-CA (Figure 3). According to the results (Table 4; Figure 5), most of the tested compounds might act as univalent DTIs, as they bind to unliganded and liganded forms of thrombin only within the active site, near Ser205 and His43 of the catalytic triad. Argatroban, the drug approved as an alternative antithrombotic treatment for patients with heparin-induced thrombocytopenia (HIT), as well as for patients undergoing percutaneous coronary interventions with or at risk for HIT, presents a similar mechanism of action–it interacts reversibly with the catalytic triad of thrombin (Marchi and López, 2016). Some of the tested blackthorn flower constituents, such as PA2, AFZ and KT, have additional binding sites outside the active site, and could be considered bivalent DTIs. For instance, PA2 has been shown to interact with the active site and exosite I. Exosite I, composed of Lys21, Arg 62, Arg 68, Arg70, Tyr71, Arg 73, Lys 106 and Lys 107 residues, is responsible for the binding of the crucial substrate for thrombin: fibrinogen and other molecules such as protease-activated receptor 1 (PAR-1), factor V, factor VIII, thrombomodulin, platelet transmembrane glycoprotein (GPIba), and hirudin (Crawley et al., 2007). Hirudin, a polypeptide derived from the saliva of the leech *Hirudo medicinalis*, forms a biomolecular complex with thrombin via its acidic *C*-terminus and thereby antagonizes this serine proteinase activity. In the last few years, several new synthetic anticoagulants based on the structure of hirudin have been introduced to therapy, for example, bivalirudin, which joins the exosite I, as well as the active site of thrombin (Marchi and López, 2016). Therefore, PA2, with a similar mechanism of action, seems to be a promising molecule in the context of new drug development. Two kaempferol glycosides, AFZ and KT, have additional binding sites located on the opposite side of the thrombin molecule relative to the exosite I. Interestingly, none of the investigated *P. spinosa* polyphenols have been shown to join the exosite II. Exosite II, composed of Arg 89, Arg 98, Arg 245, Lys 248, and Lys 252 residues, binds the anticoagulant polysaccharide heparin and platelet transmembrane glycoprotein (GPIba) and is required for the recognition and cleavage of factor V and factor VIII (Marchi and López, 2016).

Among the *P. spinosa* flower constituents, PA2, QU, KA, and *p*-cJu, represent the most significant potential for thrombin inhibition. They not only bound firmly to the active site of the enzyme as demonstrated by docking calculations with their lowest free binding energy (Table 4) but also independently inhibit thrombin amidolytic activity *in vitro* (Table 5) and showed the most substantial effect in changing the secondary structure of thrombin within the α-helices, as can be seen from CD spectra (Figure 6). CD spectroscopy has a wide range of applications, among which investigating the secondary structure of biomolecules, including proteins, is the most notable. The method quickly analyzes the conformational changes of a protein caused by the addition of ligands and is often used to evaluate the interaction of plant metabolites and enzymes (Oluwagunwa et al., 2021; Yu et al., 2022). Since the vicinity of the catalytic site and exosite I of thrombin contains several α-helices, the changes in CD spectra in the presence of blackthorn flower polyphenols may indicate their actual interaction with the enzyme by binding at these sites. PA2, due to its strong docking binding in the active site and exosite I, and the most extensive changes in CD spectra, may be an essential compound responsible for the thrombin-inhibiting effect observed for the *P. spinosa* flower extracts. Previous studies concerning the influence of PA2 on the hemostatic system established that PA2 slightly increased TT (Owczarek et al., 2021). To the best of our knowledge, the present work is the first report indicating the thrombin-inhibitory activity of PA2 and explaining the molecular mechanism of this action.

The literature data on polyphenols suggests these compounds strongly interact with thrombin. Rodrigues et al. (2015) revealed that flavonol glycosides, i.e., quercetin 3-*O*-arabinoside and quercetin 3-*O*-rhamnoside, isolated from the leaves of white mangrove (*Laguncularia racemosa*) are potent inhibitors of the enzyme. The study conducted by Bijak et al. (2014) pointed out that catechin, epicatechin, cyanidin, cyanidin 3-glucoside and quercetin had an inhibitory effect on the amidolytic activity of thrombin; however, only cyanidin, quercetin and silybin also inhibited the thrombin-induced polymerization of fibrinogen. Liu et al. (2010) reported a strong interaction between thrombin and gallic acid, catechin, epicatechin, dihydroquercetin, naringenin, apigenin, and baicalein. Choi et al. (2015) revealed that kaempferol significantly inhibited the amidolytic activity of thrombin and fibrin polymer formation. The study performed by Wei et al. (2019) documented that flavonol glycosides rutin and isoquercetin displayed moderate inhibition on the proteolytic activity of thrombin (the IC_50_ 34.08 and 28.76 µM, respectively), while the activity of quercetin and kaempferol was weaker (the IC_50_ 57.77 and 59.99, respectively). Among 42 flavonoids tested by Wang et al. (2020) in terms of their inhibition of amidolytic activity of thrombin, myricetin exhibited the highest inhibitory potential against the enzyme with the IC_50_ value of 56 μM; for comparison, the IC_50_ values of kaempferol and quercetin were 107 and 205 μM, respectively. The results from the present *in vitro* study may lead to some conclusions about the flavonoid structure-activity relationship. Among 8 flavonoid constituents tested for thrombin inhibition were aglycones (QU, KA) and their glycosides (mono- and diglycosides), including compounds with the *p*-coumaroyl group in the sugar moiety. Regarding the aglycones, the presence of the *ortho*-dihydroxyphenyl structure had a positive impact on the investigated activity. With two hydroxyl groups at 3′and 4′of the B ring, QU presented a more potent thrombin inhibition than KA with one hydroxyl group (IC_50_ 29.35 and 81.12 μg/mL, respectively). On the other hand, glycosylation limited the activity of flavonoids, as no inhibitory effects on thrombin were found for all tested glycosides at 1–50 μg/mL. The only exception was *p*-cJU, which, contrary to JU, inhibited the enzyme, and its activity was even more than twice as high as that of KA (IC_50_ 38.29 and 81.12 μg/mL, respectively). Thus, *p*-coumaroyl substitution at the 2″-OH position of arabinofuranose remarkably enhances the anticoagulant effect, and this functionalization in a sugar moiety could be regarded as a potential approach for improving the inhibitory effects of flavonoid glycosides on thrombin. Our results are consistent with that of Liu et al. (2010), which evaluated the thrombin inhibition by flavonoids using the optimized TT assay *in vitro*. As in our study, quercetin was a more potent thrombin inhibitor (the IC_50_ of 35 µM) than kaempferol (the IC_50_ of 109 µM), and the glycosides, i. a., rutin, hyperoside, and kaempferol-3-*O*-glucoside, were inactive in the tests with the IC_50_ above 1000 μM, while kaempferol-3-*O*-(*2″*-di-*E-p*-coumaroyl)-rhamnoside and kaempferol-3-*O*-(*2″,4″*-di-*E-p*-coumaroyl)-rhamnoside displayed moderate activity with the IC_50_ of 83 and 52 μM, respectively. All these data suggest that glycosylation significantly disfavors the thrombin inhibitory effect of flavonoids; however, the presence of other groups in the sugar moiety, for example, *p*-coumaroyl, prompts inhibition of the enzyme.

Polyphenols undergo extensive metabolism in the human body, and their biological *in vivo* effects stem from both their native forms and biotransformation products, such as glucuronidated, methylated, and sulfated forms (Serreli and Deiana, 2019). Some polyphenolic compounds may be absorbed but most of them reach the colon, where gut microbiota contribute to their conversion into low-molecular-weight phenolic acids with potential biological activity (Catalkaya et al., 2020). The primary gut microbiota metabolites of flavonols are DCA, PAA and PCA (Marín et al., 2015). Proanthocyanidins are metabolized into PPA, 3-(3′-hydroxyphenyl) propionic acid, 4-hydroxyphenylacetic acid, and phenylvalerolactone (Ou and Gu, 2014). CHA is metabolized by gut microbiota to caffeic acid, which is then transformed into phenylpropionic acid derivatives like DCA and PPA (Tomas-Barberan et al., 2014). For a thorough assessment of the plant materials’ biological effects, our *in silico* docking simulations considered key phenolic compounds representing the main metabolites of polyphenols in the human body and corresponding to the phenolic profile of the blackthorn flower extracts. These included miquelianin, a product of quercetin glucuronidation, as well as DCA, PCA, PPA, and PAA, which serve as model gut microbiota metabolites (Figure 4). The results revealed that the metabolized polyphenols bind to the active site of thrombin and may act as univalent direct thrombin inhibitors. In the last years, the antioxidant, anti-inflammatory and anticancer effects of microbiota metabolites have been revealed in numerous studies (Serreli and Deiana, 2019). Our previous works also revealed the significant activity of the metabolized polyphenols towards multiple *in vivo*-relevant oxidants (Marchelak et al., 2019), as well as adequate protection of human plasma components, including fibrinogen, against ONOO^−^-induced damage (Marchelak et al., 2021).

## 5 Conclusion

This paper advances the current understanding of the biological activity of blackthorn flowers by presenting new data on their impact on various aspects of the hemostatic system *in vitro*. For the first time, the results demonstrate that blackthorn flower extracts at *in vivo-*relevant levels might exert anticoagulant effects through direct thrombin inhibition. Among the extracts, MED and its polyphenolic-rich fractions DEF, EAF, and BF exhibit superior activity parameters and appear to be the most promising candidates for functional applications. As indicated by correlation studies, polyphenols significantly contribute to the thrombin-inhibitory effects of the extracts. *In silico* docking simulations suggest that blackthorn flower constituents can act as univalent or bivalent DTIs, as they bind with considerable binding affinity to thrombin only within the active site or have additional binding sites outside the catalytic triad, i.e., exosite I. The interaction of blackthorn polyphenols with the enzyme was confirmed in CD spectroscopy, which revealed changes in the secondary structure of thrombin within the α-helices. Among the individual constituents, PA2, QU, KA, and *p*-cJu demonstrate the most significant potential for antagonizing the enzyme. Notably, PA2 stands out due to strong docking binding in the active site and exosite I, extensive changes in CD spectrum, and significant thrombin inhibition *in vitro*. Additionally, our findings suggest potential structural modifications, such as *p*-coumaroyl substitution at the 2″-OH position in a sugar moiety, as an approach to enhance the thrombin-inhibitory activity of flavonoid glycosides. Moreover, our study indicates that the extracts do not significantly impact platelet hemostasis. These findings contribute to the understanding of the potential medical applications of *P. spinosa* flowers in preventing and treating cardiovascular diseases (CVDs). However, additional research is needed to thoroughly understand their impact on the hemostatic system, including their interactions with other coagulation cascade factors and fibrinolytic proteins. Furthermore, it is essential to complement *in vitro* tests with animal studies and clinical trials to finally assess the therapeutic effectiveness of blackthorn flowers and extracts.

## Figures and Tables

**Table:** 

**AFZ**	Kaempferol 3-*O*-*α*-L-rhamnopyranoside (afzelin)
**APPT**	activated partial thromboplastin time
**AQ**	aqueous extract
**AV**	Quercetin 3-*O*-*α*-L-arabinofuranoside (avicularin)
**BF**	*n*-butanol
**CA**	(+)-Catechin
**CCHA**	4-*O*-Caffeoylquinic acid (cryptochlorogenic acid)
**CFA**	Caffeic acid
**CHA**	5-*O*-Caffeoylquinic acid (chlorogenic acid)
**CVDs**	cardiovascular diseases
**DCA**	3-(3′,4′-dihydroxyphenyl)propionic acid (dihydrocaffeic acid)
**DEF**	diethyl ether fraction
**DTI**	direct thrombin inhibitors
**EAF**	ethyl acetate fraction
**ECA**	(−)-Epicatechin
**GU**	Quercetin 3-*O*-*α*-L-arabinopyranoside (guaiaverin)
**HY**	Quercetin 3-*O*-*β*-D-galactopyranoside (hyperoside)
**IQ**	Quercetin 3-*O*-*β*-D-glucopyranoside (isoquercitrin)
**JU**	Kaempferol 3-*O*-*α*-L-arabinofuranoside (juglanin)
**KA**	Kaempferol
**KAFR**	Kaempferol 3-*O*-*α*-L-arabinofuranoside-7-*O-α*-L-rhamnopyranoside
**KAPR**	Kaempferol 3-*O*-*α*-L-arabinopyranoside-7-*O-α*-L-rhamnopyranoside
**KCAR**	Kaempferol 3-*O*-(2*″*-*O*-*E*-*p*-coumaroyl)-*α*-L-arabinofuranoside-7-*O*-*α*-L-rhamnopyranoside
**KR**	Kaempferol 7-*O*-*α*-L-rhamnopyranoside
**KRG**	Kaempferol 3-O-(6″-O-α-L-rhamnopyranosyl)-β-D-glucopyranoside
**KT**	Kaempferol 3,7-di-*O*-*α*-L-rhamnopyranoside (kaempferitrin)
**KX**	Kaempferol 3-*O*-*β*-D-xylopyranoside
**LEP**	Kaempferol 3-*O*-*β*-D-xylopyranoside-7-*O*-*α-*L-rhamnopyranoside (lepidoside)
**MED**	methanol-water (7:3, v/v) extract
**MQ**	Quercetin 3-*O-β*-D-glucuronopyranoside (miquelianin)
**MUA**	Quercetin 3-O-(4″-O-β-D-glucopyranosyl)-α-L-rhamnopyranoside (multinoside A)
**MUB**	Kaempferol 3-*O*-(4*″*-*O*-*β*-D-glucopyranosyl)-*α*-L-rhamnopyranoside (multiflorin B)
**NCHA**	3-*O*-Caffeoylquinic acid (neochlorogenic acid)
**PA2**	proanthocyanidin A2
**PAA**	2-(3′,4′-dihydroxyphenyl) acetic acid
***p*-CA**	*p*-Coumaric acid
***p*-CJU**	Kaempferol 3-*O*-(2*″*-*O*-*E*-*p*-coumaroyl)-*α*-L-arabinofuranoside
**PBMCs**	peripheral blood mononuclear cells
**PCA**	protocatechuic acid
**PPA**	3-(4′-hydroxyphenyl)propionic acid
**PT**	prothrombin time
**RBCs**	red blood cells
**RN**	Quercetin 3-*O*-*α*-D-xylopyranoside (reinutrin)
**ROS/RNS**	reactive oxygen/nitrogen species
**RT**	Quercetin 3-*O*-(6*″*-*O*-*α*-L-rhamnopyranosyl)-*β*-D-glucopyranoside (rutin)
**QC**	Quercetin 3-*O*-*α*-L-rhamnopyranoside (quercitrin)
**QGA**	Quercetin 3-*O*-(2*″*-*O*-*β*-D-glucopyranosyl)-*α*-L-arabinofuranoside
**QU**	Quercetin
**TPA**	total proanthocyanidin content in cyanidin chloride equivalents (CyE) determined by *n*-butanol/HCl assay
**TPC**	total phenolic content in gallic acid equivalents (GAE) determined by Folin-Ciocalteu assay
**TPH**	total phenolic content determined by RP-HPLC-PDA as a sum of individual compounds
**TT**	thrombin time
**WR**	water residues

## Data Availability

The original contributions presented in the study are included in the article/Supplementary Material, further inquiries can be directed to the corresponding author.
