# Supplementary material for: Flavonol and A-type procyanidin-rich extracts of Prunus spinosa L. flower exhibit anticoagulant activity through direct thrombin inhibition, but do not affect platelet aggregation in vitro
Source: Front Pharmacol. 2023 Nov 27;14:1307373. doi: 10.3389/fphar.2023.1307373 (PMC10711620; doi:10.3389/fphar.2023.1307373)
Supplement: Supplementary file 1 [file Table1.DOCX]

Supplementary Material

**Flavonol and A-type procyanidin-rich extracts of *Prunus spinosa* L. flower exhibit anticoagulant activity through direct thrombin inhibition, but do not affect platelet aggregation *in vitro***

**Anna Marchelak^1^, Joanna Kolodziejczyk-Czepas^2^, Michał B. Ponczek^2^, Oleksandra Liudvytska^2^, Magdalena Markowicz-Piasecka^3^, Beata Bielska^4^, Katarzyna Miłowska^4^, Monika A. Olszewska^1^**

^1^Department of Pharmacognosy, Faculty of Pharmacy, Medical University of Lodz, Lodz, Poland;

^2^Department of General Biochemistry, Faculty of Biology and Environmental Protection, University of Lodz, Lodz, Poland;

^3^Department of Applied Pharmacy, Faculty of Pharmacy, Medical University of Lodz, Lodz, Poland;

^4^Department of General Biophysics, Faculty of Biology and Environmental Protection, University of Lodz, Lodz, Poland.

*** Correspondence:**Anna Marchelak
[anna.marchelak@umed.lodz.pl](mailto:anna.marchelak@umed.lodz.pl)

# Table of Contents

# Supplementary Table 1. UHPLC-PDA-ESI-MS^3^ data of polyphenols detected in the source aqueous (AQ) and hydroalcoholic (methanol-water, 7:3, *v/v*) (MED) extracts of *P. spinosa* flowers.

# Supplementary Table 2. Quantitative profile of the dry extracts/fractions obtained from *P. spinosa* flower (mg/g dw).

# Supplementary Figure 1. The effects of the examined *P. spinosa* flower extracts on the lag time of the polymerisation curve.

**Supplementary Table 1**

UHPLC-PDA-ESI-MS^3^ data of polyphenols detected in the source aqueous (AQ) and hydroalcoholic (methanol-water, 7:3, v/v) (MED) extracts of *P. spinosa* flowers.

| **Peak** | **Analyte** | ***R_t_***  **(min)** | **UV λ_max_**  **(nm)** | **[M–H]^‒^**  **(m/z)** | **Fragmentary ions** | **[M‒H]^+^**  **(m/z)** | **Fragmentary ions** | **Formula** | **Extract** |
| --- | --- | --- | --- | --- | --- | --- | --- | --- | --- |
| **1** | 3-*O*-caffeoylquinic acid (neochlorogenic acid)^a^ | 6.4 | 325 | 353 | 191, 179 | 355 | 163 | C_16_H_18_O_9_ | AQ, MED |
| **2** | caffeic acid hexoside | 7.2 | 325 | 341 | 179 | 365 | 185 | C_15_H_18_O_9_ | AQ, MED |
| **3** | 3-*O*-*p*-coumaroylquinic acid | 9.0 | 310 | 337 | 163, 191 | 339 | 147 | C_16_H_18_O_8_ | AQ, MED |
| **4** | 5-*O*-caffeoylquinic acid (chlorogenic acid)^a^ | 10.3 | 325 | 353 | 191, 179 | 355 | 163 | C_16_H_18_O_9_ | AQ, MED |
| **5** | 3-*O*-feruloylquinic acid | 10.8 | 325 | 367 | 193 | 369 | 177 | C_17_H_20_O_9_ | AQ, MED |
| **6** | 4-*O*-caffeoylquinic acid (cryptochlorogenic acid)^a^ | 11.1 | 325 | 353 | 173, 191, 135 | 355 | 163 | C_16_H_18_O_9_ | AQ, MED |
| **7** | Ferrulic acid hexoside | 11.7 | 325 | 355 | 193 | 379 | 217 | C_16_H_20_O_9_ | MED |
| **8** | 5-*O*-*p*-comaroylquinic acid | 14.1 | 310 | 337 | 191, 173, 163 | 339 | 177, 147 | C_16_H_18_O_8_ | AQ, MED |
| **9** | 4-*O*-*p*-comaroylquinic acid | 15.2 | 310 | 337 | 191, 173, 163 | 339 | 177, 147 | C_16_H_18_O_8_ | AQ, MED |
| **10** | Kaempferol hexoside | 20.4 | 264, 355, 285 | 447 | 357, 327, 287 | 449 | 431, 329 | C_21_H_20_O_11_ | AQ, MED |
| **11** | (epi)catechin-A-(epi)catechin | 21.1 | 280 | 575 | 423, 289 | 577 | 559, 425, 287 | C_30_H_24_O_12_ | AQ, MED |
| **12** | isorhamnetin dihexoside | 21.5 | 270, 353 | 639 | 459, 315 | 641 | 479, 317 | C_28_H_32_O_17_ | AQ, MED |
| **13** | (epi)catechin-A-(epi)catechin | 21.9 | 280 | 575 | 539, 423, 289 | 577 | 425, 287 | C_30_H_24_O_12_ | AQ, MED |
| **14** | kaempferol dihexoside | 23.0 | 264, 355 | 609 | 447, 429, 285 | 611 | 449, 287 | C_27_H_30_O_16_ | AQ, MED |
| **15** | quercetin hexoside-pentoside | 23.9 | 265, 358 | 595 | 505, 433, 301 | 597 | 435, 303 | C_26_H_28_O_16_ | AQ, MED |
| **16** | Unknown compound | 24.1 | 280 | 433 | 287 | 435 | 289 |  | AQ, MED |
| **17** | kaempferol 3-*O*-*α*-L-arabinopyranoside-7-*O*-*α*-L-rhamnopyranoside^a^ | 24.8 | 268, 356 | 563 | 417, 285 | 565 | 419, 287 | C_26_H_28_O_14_ | AQ, MED |
| **18** | quercetin 3-*O*-*β*-D-galactoside (hyperoside)^a,^ | 25.4 | 264, 355 | 463 | 301 | 465 | 303 | C_21_H_20_O_12_ | AQ, MED |
| **19** | quercetin 3-*O*-(6″-*O*-*α*-L-rhamnopyranosyl)-*β*-D-glucopyranoside (rutin)^a^ | 25.6 | 265, 354 | 609 | 301 | 611 | 465, 303 | C_27_H_30_O_16_ | AQ, MED |
| **20** | kaemferol 3-O-*β*-D-xylopyranoside-7-O-*α*-L-rhamnopyranoside (lepidoside)^a^ | 26.5 | 265, 354 | 563 | 431, 417, 285 | 565 | 433, 287 | C_26_H_28_O_14_ | AQ, MED |
| **21** | quercetin 3-*O*-*β*-D-glucopyranoside (isoquercitrin)^a^ | 26.7 | 265, 356 | 463 | 301 | 465 | 303 | C_21_H_20_O_12_ | AQ, MED |
| **22** | quercetin 3-*O*-(2″-*O*-*β*-D-glucopyranosyl)-*α*-L-arabinofuranoside^a^ | 27.7 | 258, 354 | 595 | 433, 301 | 597 | 435, 303 | C_26_H_28_O_16_ | AQ, MED |
| **23** | quercetin 3-*O*-*α*-D-xylopyranoside (reinutrin)^a^ | 29.2 | 256, 356 | 433 | 301 | 435 | 303 | C_20_H_18_O_11_ | AQ, MED |
| **24** | quercetin 3-*O*-*α*-L-arabinopyranoside (guaiaverin)^a^ | 31.0 | 255, 355 | 433 | 301 | 435 | 303 | C_20_H_18_O_11_ | AQ, MED |
| **25** | kaempferol 3,7-di-*O*-*α*-L-rhamnopyranoside (kaempferitrin)^a^ | 33.4 | 254, 356 | 577 | 431, 285 | 579 | 433, 287 | C_27_H_30_O_14_ | AQ, MED |
| **26** | kaempferol 3-*O*-*α*-L-arabinofuranoside-7-O-*α*-L-rhamnopyranoside^a^ | 33.5 | 254, 356 | 563 | 431, 285 | 565 | 433, 287 | C_26_H_28_O_14_ | AQ, MED |
| **27** | quercetin 3-*O*-*α*-L-arabinofuranoside (avicularin)^a^ | 34.1 | 255, 355 | 433 | 301 | 435 | 303 | C_20_H_18_0_11_ | AQ, MED |
| **28** | quercetin 3-*O*-(4″-*O*-*β*-D-glucopyranosyl)-*α*-L-rhamnopyranoside (multinoside A)^a^ | 34.7 | 254, 356 | 609 | 447, 301 | 611 | 449, 303 | C_27_H_30_O_16_ | AQ, MED |
| **29** | quercetin 3-*O*-*α*-L-rhamnopyranoside (quercitrin)^a^ | 35.4 | 255, 355 | 447 | 301 | 449 | 303 | C_21_H_20_O_11_ | AQ, MED |
| **30** | kaempferol hexoside-pentoside | 36.1 | 254, 355 | 579 | 417, 285 | 581 | 419, 287 | C_26_H_28_O_15_ | AQ, MED |
| **31** | kaempferol pentoside | 36.9 | 254, 355 | 417 | 285 | 419 | 287 | C_20_H_18_O_10_ | AQ, MED |
| **32** | kaempferol 3-*O*-*β*-D-xylopyranoside^a^ | 38.3 | 255, 356 | 417 | 285 | 419 | 287 | C_20_H_18_O_10_ | AQ, MED |
| **33** | kaempferol 3-*O*-*α*-L-arabinofuranoside (juglanin)^a^ | 41.2 | 254, 356 | 417 | 285 | 419 | 287 | C_20_H_18_O_10_ | AQ, MED |
| **34** | kaempferol 3-*O*-(4″-*O*-*β*-D-glucopyranosyl)-*α*-L-rhamnopyranoside (multiflorin B)^a^ | 41.6 | 256, 354 | 593 | 285 | 595 | 433, 287 | C_27_H_30_O_15_ | AQ, MED |
| **35** | kaempferol 3-*O*-*α*-L-rhamnopyranoside (afzelin)^a^ | 42.6 | 256, 356 | 431 | 285 | 433 | 287 | C_21_H_20_O_10_ | AQ, MED |
| **36** | quercetin acetyl-hexoside-rhamnoside | 45.6 | 255, 356 | 651 | 609, 447, 301 | 653 | 449, 413, 303 | C_29_H_32_O_17_ | AQ, MED |
| **37** | quercetin^a^ | 48.4 | 255, 356 | 301 | - | 303 | - | C_15_H_10_O_7_ | AQ, MED |
| **38** | kaempferol acetyl-hexoside-rhamnoside | 49.9 | 255, 355 | 635 | 593, 285 | 637 | 619, 415, 329, 287 | C_29_H_32_O_16_ | AQ, MED |
| **39** | kaempferol 7-*O*-*α*-L-ramnopyranoside^a^ | 50.1 | 254, 356 | 431 | 285 | 433 | 287 | C_21_H_20_O_10_ | AQ, MED |
| **40** | unknown compound | 51.1 | 310 | 614 | 452, 358, 316 | 616 | 454, 436 |  | AQ, MED |
| **41** | kaempferol^a^ | 54.0 | 255, 356 | 285 | - | 287 | - | C_15_H_10_O_6_ | AQ, MED |
| **42** | kaempferol 3-*O*-(2″-*O*-*E*-*p*-coumaroyl)-*α*-L-arabinofuranoside-7-*O*-*α*-L-rhamnopyranoside^a^ | 54.1 | 267, 316, 355 | 709 | 563, 285 | 711 | 565, 279 | C_35_H_34_O_16_ | AQ, MED |
| **43** | quercetin *p*-coumaroyl-pentoside | 55.6 | 267, 316, 355 | 579 | 433, 301 | 581 | 279 | C_29_H_24_O_13_ | AQ, MED |
| **44** | kaempferol 3-*O*-(2″-*O*-*E*-*p*-coumaroyl)-*α*-L-arabinofuranoside^a^ | 57.3 | 267, 316, 355 | 563 | 285 | 565 | 279 | C_29_H_24_O_12_ | AQ, MED |

*^a^*Identified with authentic standards. *R_t_*, retention times. UV λ_max_, absorbance maxima in PDA spectra. [M‒H]^‒^, pseudomolecular ions in MS spectra recorded in a negative mode. [M‒H]^+^, pseudomolecular ions in MS spectra recorded in a positive mode.

**Supplementary Table 2**

Quantitative profile of the dry extracts/fractions obtained from *P. spinosa* flower (mg/g dw).

| Analyte | Content (mg/g dw) | | | | | |
| --- | --- | --- | --- | --- | --- | --- |
|  | AQ | MED | DEF | EAF | BF | WR |
| Individual compounds: |  |  |  |  |  |  |
| **NCHA** | 4.89 ± 0.14*^D^* | 14.46 ± 0.23*^B^* | nd. | 3.04 ± 0.05*^E^* | 27.02 ± 0.37*^A^* | 10.83 ± 0.03*^C^* |
| **CHA** | 3.83 ± 0.07*^C^* | 5.64 ± 0.11*^B^* | nd. | 5.69 ± 0.32*^B^* | 15.43 ± 0.11*^A^* | 2.02 ± 0.02*^D^* |
| **CA** | < LOQ | < LOQ | 5.55 ± 0.14*^A^* | nd. | nd. | nd. |
| **CCHA** | 4.51 ± 0.06*^B^* | 4.26 ± 0.07*^C^* | nd. | 2.10 ± 0.08*^E^* | 10.56 ± 0.11*^A^* | 3.06 ± 0.01*^D^* |
| **CFA** | < LOQ | < LOQ | 7.65 ± 0.18*^A^* | nd. | nd. | nd. |
| **ECA** | < LOQ | < LOQ | < LOQ | nd. | nd. | nd. |
| **KAPR** | < LOQ | 2.69 ± 0.10*^B^* | nd. | 1.91 ± 0.08*^C^* | 10.47 ± 0.13*^A^* | nd. |
| **LEP** | 1.21 ± 0.04*^D^* | 3.17 ± 0.09*^C^* | nd. | 4.85 ± 0.14*^B^* | 10.92 ±0.19*^A^* | nd. |
| **pCA** | < LOQ | < LOQ | 8.24 ± 0.22*^A^* | nd. | nd. | nd. |
| **KT** | 5.65 ± 0.06*^E^* | 17.42 ± 0.79*^C^* | 6.13 ± 0.30*^D^* | 41.46 ± 0.19*^B^* | 48.75 ± 0.03*^A^* | nd. |
| **KAFR** | 5.15 ± 0.10*^D^* | 15.13 ± 0.21*^C^* | 0.95 ± 0.02*^E^* | 41.96 ± 1.89*^A^* | 29.84 ± 0.07*^B^* | nd. |
| **RT** | 2.53 ± 0.14*^C^* | 4.65 ± 0.15*^B^* | nd. | 2.41 ± 0.06*^C^* | 16.56 ± 0.16*^A^* | nd. |
| **QGA** | 4.80 ± 0.06 | 6.28 ± 0.25*^B^* | nd. | 3.71 ± 0.02*^C^* | 25.77 ± 0.15*^A^* | nd. |
| **IQ** | 0.41 ± 0.03*^C^* | 1.33 ± 0.03*^B^* | nd. | 8.46 ± 0.14*^A^* | nd. | nd. |
| **HY** | 0.74 ± 0.03*^C^* | 0.92 ± 0.04*^B^* | nd. | 4.05 ± 0.15*^A^* | nd. | nd. |
| **KRG** | 1.22 ± 0.12*^D^* | 3.67 ± 0.14*^C^* | nd. | 5.40 ± 0.11*^B^* | 14.10 ± 0.31*^A^* | nd. |
| **RN + GU** | 2.26 ± 0.09*^D^* | 4.26 ± 0.15*^C^* | 8.50 ± 0.32*^B^* | 18.75 ± 0.05*^A^* | nd. | nd. |
| **MUA** | 3.71 ± 0.04*^D^* | 5.38 ± 0.20*^C^* | nd. | 7.22 ± 0.34*^B^* | 19.56 ± 0.06*^A^* | nd. |
| **AV** | 8.59 ± 0.11*^D^* | 14.89 ± 0.65*^C^* | 71.04 ± 2.42*^A^* | 28.81 ± 1.12*^B^* | nd. | nd. |
| **QC** | 3.37 ± 0.09*^D^* | 7.41 ± 0.21*^C^* | 22.11 ± 0.35*^B^* | 34.41 ± 1.23*^A^* | nd. | nd. |
| **KX** | 1.73 ± 0.05*^D^* | 2.97 ± 0.10*^C^* | 16.85 ± 0.65*^A^* | 10.23 ± 0.29*^B^* | nd. | nd. |
| **MUB** | 6.63 ± 0.10*^D^* | 8.82 ± 0.07*^C^* | nd. | 24.52 ± 0.80*^A^* | 19.57 ± 0.07*^B^* | nd. |
| **JU** | 8.61 ± 0.15*^D^* | 13.73 ± 0.43*^C^* | 96.14 ± 1.33*^A^* | 16.90 ± 0.26*^B^* | nd. | nd. |
| **AFZ** | 5.65 ± 0.15*^D^* | 13.33 ± 0.16*^C^* | 115.46 ± 3.98*^A^* | 43.37 ± 1.89*^B^* | nd. | nd. |
| **KR** | 0.60 ± 0.06*^E^* | 1.78 ± 0.04*^D^* | 16.41 ± 0.16*^A^* | 9.29 ± 0.21*^B^* | 2.41 ± 0.05*^C^* | nd. |
| **KCAR** | < LOQ | 1.47 ± 0.02*^C^* | 7.37 ± 0.34*^A^* | 4.24 ± 0.23*^B^* | nd. | nd. |
| **QU** | 0.57 ± 0.03*^D^* | 1.32 ± 0.06*^C^* | 42.92 ± 1.09*^A^* | 20.99 ± 0.50*^B^* | nd. | nd. |
| **KA** | 0.72 ± 0.02*^D^* | 1.06 ± 0.01*^C^* | 41.08 ± 1.15*^A^* | 9.28 ± 0.34*^B^* | nd. | nd. |
| **pCJU** | 0.35 ± 0.02*^C^* | 1.43 ± 0.03*^B^* | 25.32 ± 0.64*^A^* | nd. | nd. | nd. |
| Phenolic fractions: |  |  |  |  |  |  |
| **TPH** | **77.71** | **157.47** | **491.69** | **353.07** | **250.95** | **15.91** |
| **TPC** | **126.98 ± 1.61*^E^*** | **206.07 ± 10.86*^D^*** | **464.57 ± 20.57*^B^*** | **584.07 ± 12.98*^A^*** | **296.57 ± 3.28*^C^*** | **64.6 ± 1.93*^F^*** |
| **TPA** | **24.46 ± 1.26*^C^*** | **45.13 ± 2.38*^B^*** | **49.5 ± 2.23*^B^*** | **109.43 ± 3.71*^A^*** | **46.6 ± 1.14*^B^*** | **12.43 ± 0.25*^D^*** |

The results are presented as means ± SD (*n* = 3). Different superscripts in each row indicate significant differences in the means at *p* < 0.05. The quantitative profile of MED and its fractions according to Marchelak et al.2017, 2020a, 2020b.

A

**Supplementary Figure 1.** The effects of the examined *P. spinosa* flower extracts on the lag time of the polymerisation curve. A – study on the isolated fibrynogen; B – study on the blood plasma. Results are presented as means ± SE (*n* = 6). Statistical differences: ** *p* < 0.01, and *** *p* < 0.001 for samples in the presence of the analytes (1, 5, 50 μg/mL) versus control samples.
